# A CI-Independent Form of Replicative Inhibition: Turn Off of Early Replication of Bacteriophage Lambda

**DOI:** 10.1371/journal.pone.0036498

**Published:** 2012-05-10

**Authors:** Sidney Hayes, Monique A. Horbay, Connie Hayes

**Affiliations:** Department of Microbiology and Immunology, College of Medicine, University of Saskatchewan, Saskatoon, Canada; Niels Bohr Institute, Denmark

## Abstract

Several earlier studies have described an unusual exclusion phenotype exhibited by cells with plasmids carrying a portion of the replication region of phage lambda. Cells exhibiting this inhibition phenotype (IP) prevent the plating of homo-immune and hybrid hetero-immune lambdoid phages. We have attempted to define aspects of IP, and show that it is directed to *rep*λ phages. IP was observed in cells with plasmids containing a λ DNA fragment including *oop*, encoding a short OOP micro RNA, and part of the lambda origin of replication, *ori*λ, defined by iteron sequences ITN1-4 and an adjacent high AT-rich sequence. Transcription of the intact *oop* sequence from its promoter, *p_O_* is required for IP, as are iterons ITN3–4, but not the high AT-rich portion of *ori*λ. The results suggest that IP silencing is directed to theta mode replication initiation from an infecting *rep*λ genome, or an induced *rep*λ prophage. Phage mutations suppressing IP, i.e., Sip, map within, or adjacent to *cro* or in *O*, or both. Our results for plasmid based IP suggest the hypothesis that there is a natural mechanism for silencing early theta-mode replication initiation, *i.e.* the buildup of λ genomes with *oop* ^+^ *ori*λ^+^ sequence.

## Introduction

**Figure 1:**
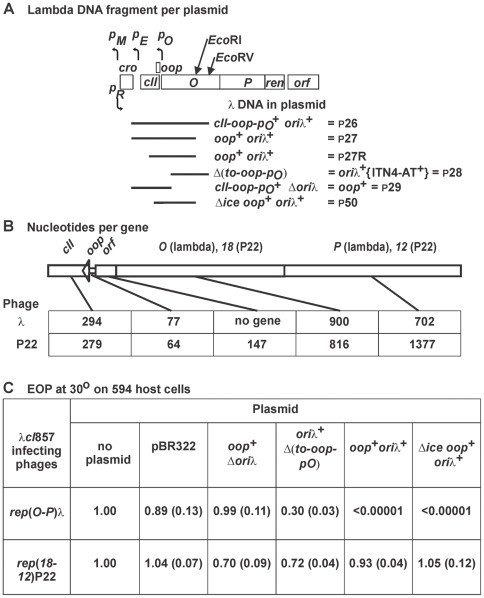
Replication-targeted inhibition of *rep*λ phage plating. A. Plasmid cloned λ DNA fragments used to map the sequence requirement(s) for an inhibition phenotype (IP). B. Genomic region spanning five contiguous and partially homologous genes of phages λ and P22 (see Fig. S2). Phage λ is naturally missing the *orf48* gene between *oop* and *O* that is present between *oop* and *18* in P22 [37], [51]. C. Assay for EOP, defined as phage titer on strain 594 (with one of the indicated plasmids) / titer on 594 cells, where plating on 594  =  EOP of 1.0. All of the plasmids shown were derived from pBR322. The *oop^+^ ori*λ^+^ plasmid used was p27. The DNA substitution of the “*ice*” [16] sequence of λ to make plasmid Δ*ice oop* ^+^ *ori*λ^+^ ( =  p50) is shown in Fig. S3A. Numbers in brackets represent standard error values.

Normal cellular immunity to λ infection arises upon the lysogenic conversion of *E. coli* cells by a λ prophage. The CI repressor protein encoded by the prophage binds to the *o_L_* and *o_R_* operator sites, each with three repressor binding sites, e.g., *o_R_*3, *o_R_*2, *o_R_*1, within the *imm*λ gene cluster *p_L_-o_L_*-*rexB-rexA-cI-p_M_*-*o_R_*-*p_R_-cro*. CI protein within a λ lysogenic cell blocks transcription of the phage genes situated downstream from the major leftward and rightward phage promoters *p_L_* and *p_R_* [1], both from the resident prophage, or when a homo-immune *imm*λ phage infects the cells. The variant λvir efficiently forms plaques on cells lysogenized by λ because it carries point mutations v2 in *o_L_*, v1 in *o_R_*2, and v3 in *o_R_*1[2]. Transcription from *p_R_* (Fig. 1A) is required for expression of genes *cro-cII-O-P*, respectively encoding a second repressor (Cro) that binds to *o_R_*; an unstable stimulator (CII) of the establishment mode of *cI* transcription from promoter *p_E_* [3]; and the *rep*λ replication initiation cassette including genes *O, P*, and the origin (*ori*λ within *O*) site, which participate in *ori*λ-dependent bidirectional (theta mode) replication initiation. The gene *oop*, is transcribed from promoter *p_O_* [4] (opposite orientation from *p_R_*), partially overlaps the terminal end of *cII*, and encodes a short self-terminating antisense RNA (OOP) opposing CII expression [5]. Part of *oop* and *p_O_* share a 33 bp region of high sequence homology within lambdoid phages (Fig. S1). The organizational similarity within the region encoding the *cII*-like–*oop*–“orf”–*O*-like–*P*-like genes for lambdoid phages is shown in Fig. S2.

The dual infection of a λ lysogen with two phages, a homo-immune *imm*λ phage and a hybrid hetero-immune λ*imm*434 phage, each of which share an identical *rep*λ replication initiation cassette, revealed that the *imm*434 phage predominated by 20^+^-fold over the *imm*λ phage in the cell burst [6]. The impaired replication of the homo-immune *imm*λ phage, described as *replicative inhibition*, which we consider herein “CI-dependent” was explained by the assumption that CI repressor molecules made by the λ prophage in the co-infected lysogenic cells prevented replication of the homo-immune phage, even when the λ replication initiation proteins (gpO and gpP) were provided *in trans* by the hetero-immune phage. The observations that CI-dependent replicative inhibition was suppressed by mutations in *o_R_* causing *p_R_* to become insensitive to repression, or by base changes creating new promoter sites downstream from *p_R_*, as exemplified by *c*17 and four *ri* ^c^ (replication inhibition constitutive) mutations [7], provided support for an argument that transcription from *p_R_* (*transcriptional activation*) was required *in cis* for theta-mode replication initiation, and that replicative inhibition was explained by CI repressor in the lysogen preventing transcriptional activation of replication initiation from the co-infecting *imm*λ *rep*λ phage.

Plasmids termed λdv were derived from phage λvir [8], [9]. They encode the *imm*λ and *rep*λ regions and are capable of autonomous replication. Early studies with cells transformed with λdv suggested that the cells acquired an unusual immunity or exclusion phenotype [8], [10] and inhibited plating by homo-immune phages, including λvir, and hetero-immune hybrid phages as λ*imm*434. Some other hetero-immune phages (*e.g.*, λ*imm*21 and λ*imm*80) that were presumably *rep*λ were able to escape the inhibition, i.e., could plate efficiently on cells transformed with λdv [8], [10]. The ability of cells with λdv plasmids to inhibit λvir development was rationalized by the suggestion that cells with this plasmid make more CI repressor than would a cell with a single λ prophage, and the higher levels of repressor would eventually bind the altered λvir operators [8]. However, CI levels were not actually measured. No explanation was provided for the inhibition of λ*imm*434 development. When RNA transcription levels from cells with λdv1 plasmid were measured, it was found that little [10] or no [11] *cI* transcription was detected, showing that the inhibition of homo-immune infecting phage development by λdv plasmid was not due to CI repressor activity. It was proposed [10] that the λdv-mediated inhibition of infecting *rep*λ phage development represents a competition for bacterial protein(s) between the plasmid and an infecting phage, and that the site for the competition was different in the λ*imm*21 and λ*imm*80 phages that escaped the inhibition.

Independently, Rao and Rogers [12] demonstrated that cells containing a pBR322/λ hybrid plasmid that included the *imm*λ and *rep*λ regions exhibited an *inhibition phenotype* (referred to herein as “IP”), that prevented the plating of λvir and λ*imm*434 infecting phage, but allowed λ*imm*21 to plate at high EOP. They reported isolating mutants of λvir and λ*imm*434 which formed plaques at high EOP on cells with the plasmid, but the causative mutations were not further identified. Another inhibition phenotype, termed *nonimmune exclusion* (NIE) [13], was specific for *imm*λ and *imm*434 phages that were *rep*λ. NIE was exhibited by a variety of engineered cells with thermally induced (CI-inactivated) cryptic *cI* [Ts] prophage deleted for *attL* through *kil*, all genes rightward of P, and had acquired mutations inactivating *P* [14]. Seven independent λ se (suppress exclusion) mutations of λ wt (wild type) were isolated from NIE phenotype cells having a *cro*27 mutation in the cryptic prophage. The se defects were point mutations within *o_R_*2 (se100a, identical to mutation v1; and se101b) and within *o_R_*1 (five mutations represented by se109b, identical to mutation vC1, and at the same site as vs387) [13]. All seven λ se isolates exhibited a CI-defective phenotype, complemented for *cII* and *cIII*, and were about 10-fold less sensitive to replicative inhibition than λ wt or λ *cI* ^-^ [13].

We have attempted to understand further the inhibition phenotype(s), IP, by constructing plasmids with portions of *rep*λ. By removing *imm*λ from plasmids, the conflicting plating data for λvir was eliminated. We have shown that CI-independent, plasmid-dependent IP requires *cis* acting *ori*λ iteron (ITN) sequences [2], [15] and *oop* transcription, and is directed to *rep*λ phages. We suggest that the target of IP is early (theta-mode) replication initiation. Phage mutations suppressing IP, i.e., Sip, map within, or adjacent to *cro* or in *O*, or both.

## Results

### Plasmid-mediated Inhibition Phenotype (IP)

The bacterial strains, phage, plasmids and primers for modifying plasmids are described in Tables 1, 2, 3. Plasmid pCH1, theoretically identical to the IP plasmid described by Rao and Rogers [12], and deletion derivatives as p25 and others (Table 2, Fig. 1A) were made to determine which λ sequences were responsible for IP. Plasmids pCH1 and p25 inhibited the plating of λvir, but versions deleting *imm*λ (including the *p_R_* promoter) did not (data not shown). The IP toward *rep*λ phage was seen with plasmids as p26 (data not shown), p27, (*rop* ^+^, *oop* ^+^, *ori*λ^+^), p27R (*oop* ^+^, *ori*λ^+^), and p50 (Δ*ice oop* ^+^ *ori*λ^+^) in Fig. 1C. p50 was deleted for the proposed replication inceptor site *ice* [16] (Fig. S3A,C,D), including all λ DNA from 31 bp leftward / downstream of the *oop* sequence (Fig. S3A). Plasmids that were *oop* ^+^ Δ*ori*λ, or *ori*λ^+^ but deleted for the *t_O_-oop-p_O_* sequence expressing the self-terminating 77 nt OOP RNA [17] (Fig. S3B), were defective in IP. In contrast, phages where *rep*λ was replaced by *rep*P22 as in λ*cI*857(*18,12*)P22 escape IP (Fig. 1C; gene replacements are shown in Fig.'s 1B, S2, S3C). These results strongly suggest that IP is directed to *rep*λ phages that employ genes *O* and *P* to initiate replication from *ori*λ.

**Table 1:** *E. coli* K12 and Bacteriophage λ Strains.

Bacteria and phages	Relevant Genotype	Hayes lab # and source
594 [70] (presumably = R594)[71]	Sup^o^ cells; F^−^ *lac*-3350 *galK*2 *galT*22 *rpsL*179 IN(*rrnD-rrnE*)1	B10 [70]; Bachmann [71]
W3350 [72] (W3350A )	Sup^o^ cells; F^−^ *lac*-3350 *galK*2 *galT*22 IN(*rrnD-rnE*)1	B12, Campbell & Balbinder, 1958, cited in [72]; Bachmann [71]
W3350 *dnaB*-GrpD55	*dnaB-*GrpD55 *malF*3089::Tn*10* Tet^R^	nB15; Bull & Hayes [36]
TC600	*thr*1 *leuB*6 *fhuA*21*lacY*1 *glnV*44 el4^-^ *glpR*200 *thi*1*supE*	B8; Bachmann [71]
Y836	Strain with cryptic λ*c* [Ts]857 prophage a	Y836; Hayes and Hayes [13], derived from strain SA431 [73]
594(λ*cI*857)^b^	*imm*λ *cI*[Ts]857 *rep*λ prophage	nY1016; this work
594(λ*cI*857(*18,12*)P22)^b^	*imm*λ *cI*[Ts]857 *rep*P22 prophage	nY1111; this work
λ*cI*857	*cI*[Ts]857 *rep*λ	1002; Hayes [49]
λ*cI*72	*cI* ^−^ *rep*λ	999; Hayes [49]
λ vir	point mutations in *o_L_*2, *o_R_*1, and *o_R_*3, *rep*λ	1000; Hayes [49]
λ*cI*857(*18,12*)P22	*imm*λ *cI*[Ts]857 *rep*P22 = λhy106	998; Hayes & Hayes [13]
λ*cI* ^+^Δ*cII*	326-bp deletion of *cII* in λ *c* ^+^	992; L. Thomason [50]
λ papa	( = wild type *cI* ^+^)	241; Hayes & Hayes [13]
λ*cI*90c17	c17, 9-bp duplication at 38341λ [2]	1006; Hayes & Hayes [13]
λse100a	*oR* 37979λ GC->TA, CI^−^ phenotype	1003; Hayes & Hayes [13]
λse101b	*oR* 37985λ CG->AT, CI^−^ phenotype	1004; Hayes & Hayes [13]
λse109b	*oR* 38009λ CG->AT, CI^−^ phenotype	1005; Hayes & Hayes [13]
λ*imm*434*cI*#5	*imm*434 *cI*	957, Hayes *et al.* [14]
λ*imm*434 Δnin5	deletion NinR recombination functions c	969; Hayes *et al.* [18]
*λbio*275 *imm434*	deletion of NinL recombination functions c	958; Hayes *et al.* [18]
*λbio*275 *imm434* Δnin5	deletion of NinL and NinR functions	952; Hayes *et al.* [18]

a The λ prophage genes *int-xis-exo-bet-gam-kil* in strain Y836 were substituted with *bio*275 [13]. The strain carries the chromosomal deletion Δ431[33] that removes genes rightward from ninB in prophage through *moaA* in host, including prophage genes *orf146* (*orf*) – *J*b2 (*i.e.*, all the late genes required for cell lysis and phage morphogenesis). A map of the cryptic lambda prophage in strain Y836 is drawn in Fig. 4A.

b Lysogenic strains show the prophage within the cell by “( )” bordering the prophage.

c The NinR region deleted by Δnin5 removes λ bases 40,503–43,307, i.e., *ren-ninA – ninI* (including *orf -ninC* and *rap-ninH* ); the NinL region substituted by *bio*275 replaces genes *int-xis-hin-exo-bet-gam-kil*, representing λ bases 27,731–∼33,303 [18].

**Table 2:** Plasmids^a^.

Plasmid	λ bases	Bases from pBR322
pCH1^b^	34500–41731	1–375, 376–4361
p25^b^	34500–39354	1–187, 376–4361
p26^b^	38215–39354	1–187, 376–4361
p27^b^	38215–39168	1426–4359
p27R^b^	38359–39168	2297–4359
p28^b^	38815–39354	1–187, 376–4361
p29^b^	38215–38835	1–187, 376–4361
p50^c^	38568–39168	188–4359
p51^c^	38568–38759, 38820–39168^d^	188–4359
p51kan^c^	38568–38759, 38814–39168^e^	188–4359
p52^c^	38568–38759, 38814–39168^f^	188–4359
p27R*p_O_*−	38359–38683, 38689–39168, bases 38684–38688 (ATTAT) replaced with GCGCG	2297–4359
p27R-R45OOP	38359–38629, 38675–39168, bases 38630–38674 substituted^g^	2297–4359
p27RΔAT	38359–39127	2297–4359
p27RΔITN1–4	38359–39043, 39120–39168	2297–4359
p27RΔITN3–4	38359–39077, 39120–39168	2297–4359
pcIpR-*O*-timm	modified 35799–35824, 37203–38036, 38686–39582^h^	1–3, 651–4361
P434'pR-*O*-timm	modified 35799–35824, 37203–37464, 38686–39582^i^	1–3, 651–4361

a All plasmids were prepared in this laboratory.

b Described in [11], some illustrated in Fig. 1A.

c Described in [37], [69], illustrated in Figures 1, S3.

d Deletes 60 bp within *O* between 38759 and 38820, include one of two *Bgl*II sites.

e ∼1450 bp *Bgl*II DNA fragment with Kan^R^ (derived from *Tn*903) within pUC4K was inserted within the remaining *Bgl*II site in p51.

f Removal of ∼1426 bp *Pst*I fragment from ∼1450 bp Kan^R^ fragment, adding 24 bp within the 60 bp deletion region between bases 38759 and 38820.

g Initially 45 random bases were chosen, but then some bases were modified to remove the possibility for secondary structure (hairpin) formation.

h Expression plasmid [74]–[76] where *O* expression is regulated by CI[Ts]857 repressor from *p_R_* promoter.

i Part of *cI*857 –*p_R_* in pcIpR-*O*-timm was replaced with 379 bp N-terminal 434-*cI*[Ts]–*p_R_* sequence, resulting in constitutive expression of *O* [75].

**Table 3:** Primers used for plasmid modification.

Plasmid	Unique Primers^a^	Sequence
p27RΔAT	LPo1	5′-CACACCGCATATGGTTCGTGCAAAC
p27RΔAT	RΔAT1	5′-AAGAATTCCTTTTGTGTCCCCCT
p27R*p_O_*−	RPo2	5′-TGCTGTATTTG TCGCGCGGACTCCTGTTGA
p27R*p_O_*−	LPo3	5′-TCAACAGGAGTCCGCGCGACAAATACAGCA
p27R*p_O_*−	RPo4	5′-AAGAATTCTCTGACGAATAATCT
p27R-R45OOP	LROOP3	5′-TAATGAGAGTATAAAAGCAAAGGGAGAGAG- ATAATAGTACAGAAGCAGGAGTCATTATGACAA
p27R-R45OOP	RROOP2	5′-CTTCTGTACTATTATCTCTCTCCTTTGCTTTT-ATACTCTCATTAAGAACGCTCGGTTGCCGC
p27RΔITN1–4	LΔITN1–4	5′-AAAACATCTCAGAATGGTGCCACAAAAGAC-ACTATTACAAAAGAA
p27RΔITN1–4	RΔITN1–4	5′-TTCTTTTGTAATAGTGTCTTTTGTGGCACCA-TTCTGAGATGTTTT
p27ΔITN3–4	LΔITN3–4	5′-CCTAAAACGAGGGATAAAACCACAAAAGA- CACTATTACAAAAGAA
p27ΔITN3–4	RΔITN3–4	5′-TTCTTTTGTAATAGTGTCTTTTGTGGTTTTA-TCCCTCGTTTTAGG

a L and R primer sequences are from the lambda *l*-strand (coding strand for *cII-O*) and *r*-strand (coding strand for *cI* and *oop*) sequences, respectively.

**Figure 2:**
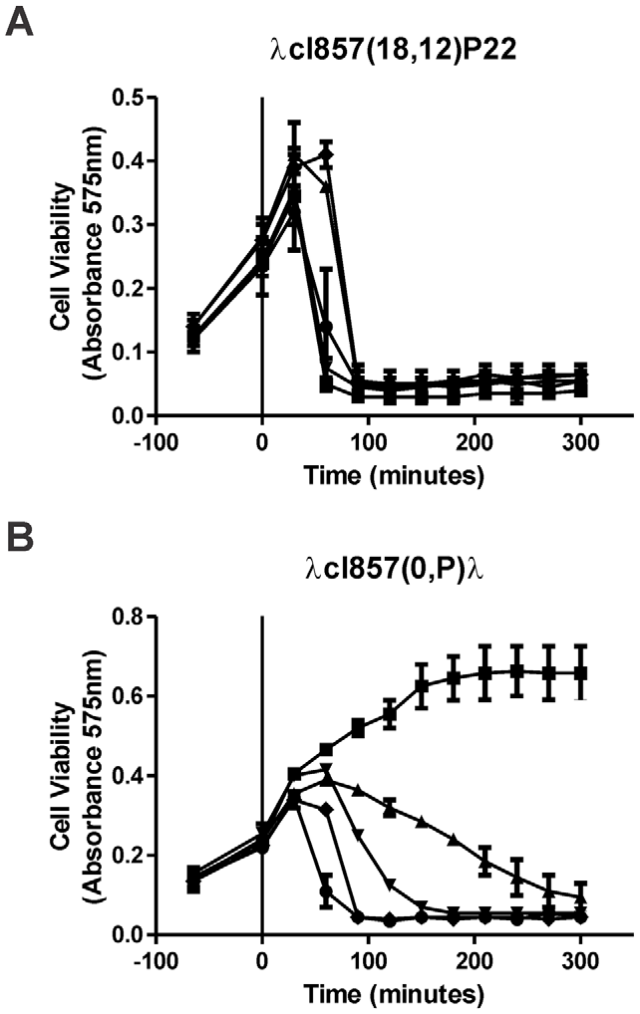
Thermal Induction of *rep*λ or the *rep*P22 –hybrid λ*cI*857 prophages. Lysogenic cultures of strain 594 were grown at 30° and each prophage was thermally induced by shifting the culture from 30° to 42° at time 0. A. Thermally induced *rep*P22 prophage. B. Thermally induced *rep*λ prophage. The results represent the averages for 2 independent assays. Plasmids within lysogenic cells: square, *Po* ^+^ *oop* ^+^ *ori* ^+^ (results shown for p27R, but identical results were observed for p27); triangle, *Po*− *oop* ^+^ *ori* ^+^; inverted triangle, Δ (*to-oop-Po*) *ori*λ^+^ (ITN-AT^+^); diamond, *cII*-*oop-Po* ^+^ Δ*ori*λ; circle, none (no plasmid). The standard deviation is shown for all of the data points, but is too small for visualization in some data intervals.

The influence of IP on the temporal events for cell lysis and phage burst following thermal induction of a prophage was examined (Fig. 2). None of the four plasmids, p27R, p27R*p_O_* ^-^ (*oop* ^+^ *p_O_* ^−^ *ori*λ^+^), p28 (*ori*λ^+^) and p29 (*t_O_-oop-p_O_* ^+^) (Fig. 2A) prevented phage-dependent cell lysis by an induced *rep*P22 prophage (Fig. 1B). In contrast, vegetative development of the *rep*λ prophage was markedly inhibited (as was cell lysis) in cells with the *oop* ^+^ *ori*λ^+^ plasmid (Fig. 2B); but, when the plasmid was altered by changing the -10 region for *p_O,_* or removing the *t_O_−oop-p_O_*, or *ori*λ regions, no inhibition of *rep*λ prophage development was observed, in agreement with the plating results in Fig. 1C.

We examined if a cloned intact *O* gene, repressed at 30°C, but expressed at 39° and 42°, exhibited IP to *rep*λ phage plating (Table 4). The result was similar to that for the Δ (*t_O_-oop-p_O_*) *ori*λ^+^ plasmid carrying a fragment of *O* (Fig. 1C), *i.e.,* no significant IP. The plasmid version containing intact *O*/*ori*λ, with *cI* from *imm*λ, reduced the plaque diameter of all four assayed *rep*λ phages but the version with a hybrid *imm*λ-imm434 *cI* gene did not. λvir was inhibited for plating at 30° in cells with multiple copies of the *O*/*ori*λ plasmid version with *cI* from *imm*λ, while λ*imm*434*cI* was not inhibited, suggesting λvir plating remains sensitive to high CI repressor concentration (we made a similar observation with another *cI* ^+^ plasmid [18]).

**Table 4:** Averaged EOP on host cells +/− plasmids with cloned *O* gene^a^.

Phage	594	594[pcIpR-*O*-timm]^b^			594[p434′pR-*O*-timm]^b^	
	(30, 39, 42°)^c^	30° d	39° d	42° d	30° e	42° e
λ*imm*434*cI*	1.0	0.25	0.30	0.65	1.0	1.0
λ*cI*72	1.0	<1×10^−8^	<1×10^−8^	0.20	0.76	0.76
λ*cI*[Ts]857	1.0	nd^f^	nd^f^	0.27	0.77	1.0
λvir	1.0	<1×10^−6^	0.75	0.74	1.0	1.0

a The average EOP per indicated phage was relative to that phage plating on strain 594 cells. The standard errors were all <0.05.

b The precise *O* sequence (ATG = 38686–39582 plus TAA stop codon that replaces normal TGA stop at end of *O*) was cloned to make plasmids pcIpR-*O-*timm and p434′pR-*O*-timm. In each plasmid, gene *O* occupies the position corresponding to λ gene *cro* (in phage) and the consensus Shine Dalgarno sequence for *cro* was maintained ahead of *O* in pcIpR-*O-*timm [75], [76]. In p434′pR-*O*-timm, the SD differed by one bp compared to the SD in pcIpR-*O-*timm because of the slightly different sequence ahead of *cro* in *imm*434 DNA [77]. The *O* gene within pcIpR-*O-*timm is transcribed from *p_R_* and regulated by *cI*[Ts]857 repressor: at 30° *O* is repressed, at 39° and 42° *O* is expressed, or fully expressed. Gene *O* is constitutively expressed from *p_R_* in p434′pR-*O*-timm.

c The column for plating at 30°, 39° and 42° yielded equivalent phage titers on 594 and the EOP was set to 1. Plaques ranged between 0.5–2 mm in diameter.

d Plaques formed were tiny.

e Plaques ranged from 0.3–1 mm diameter.

f nd is not done, since equivalent results were expected as seen for λ*cI*72.

### Dissecting IP sequence requirement(s)

**Figure 3:**
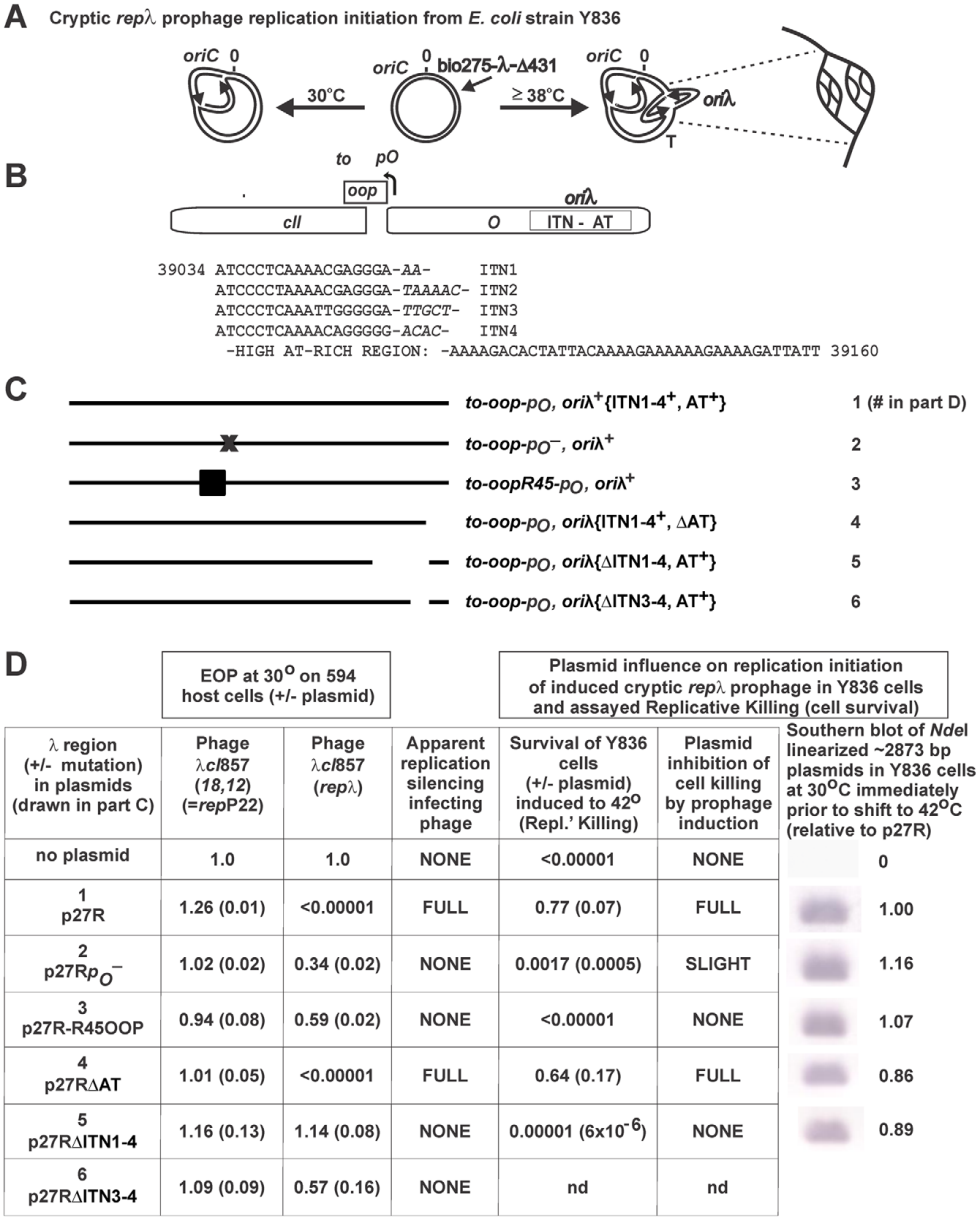
Replication silencing of *rep*λ phages requires *oop*, and iterons (**ITN**) **from** ***ori*** **λ.** A. The non-excisable cryptic λ fragment (short arrow) inserted within the *E. coli* chromosome in strain Y836 [13], [35] remains repressed at 30° where the prophage repressor is active. Shifting cells to about 39° inactivates the CI857 repressor that prevents λ prophage transcription and replication initiation from *ori*λ. Multiple λ bidirectional replication initiation events from *ori*λ generate the onion-skin replication structure drawn at right. B. Map showing *oop*-*ori*λ region. The DNA sequence for *ori*λ, shown as a rectangle around ITN-AT within gene *O* has four repeated 18 bp iteron sequences (ITN1 to ITN4), each separated by short spacer, and joined by a 38 bp high AT-rich sequence. The genes *cII* and *O* are each shown truncated and are transcribed rightward from *pR*. The *oop* sequence, which overlaps *cII* is transcribed leftward from *pO*. C. Illustrated mutations within the λ DNA region in plasmids numbered 1–6 (Table 2). Plasmid p27R (shown as #1) carries with WT sequence from which other plasmids were derived. In each plasmid the *rop* gene was deleted to provide higher plasmid copy number per cell to test the stringency of introduced mutations. The “X” in #2 inactivates the *p_O_* promoter for *oop* gene; the filled rectangle in #3 (mutation *oop*R45) substitutes random 45 bp for 45 bp within *oop* providing a 77nt RNA without internal secondary structure (Fig. S3B); and the gaps in #'s 4–6 are deletions (Table 2). D. Columns (left ‘a,’ to right ‘g’): Lane ‘a’ shows the plasmid number and common name (Table 2), with plasmid genotype indicated in part C. Lanes ‘b’ and ‘c’: EOP of repP22 and repλ phages on 594 host cells with indicated plasmid; ‘d’ summary of the inhibitory effect of a plasmid in 594 cells to the plating of repP22 or repλ phages, where NONE is essentially no inhibition of plating and FULL indicates that plaque formation was prevented by the presence of the plasmid. Lanes “e” through “g” indicate the results of a separate experiment to determine if plasmids #1–5, transformed into strain Y836, can suppress Replicative Killing, which occurs upon prophage induction when the Y836 cells are raised above 38°C. Prophage induction leads to replication initiation from *ori*λ within the chromosome, as shown in part A, which is very lethal to cell. Lane ‘e’ shows the level of cell survival upon shifting the cells to 42°C. The survival of Y836 cells that were diluted and spread on plates incubated at 42°C requires plasmid suppression / interference of replication initiation and cell killing upon de-repression of the prophage in Y836 cells. Two single colonies of each transformant of Y836 cells were inoculated into 20 ml TB +50 ug/ml ampicillin and grown overnight at 30°C. The following day the cultures were subcultured (2.5 ml overnight culture +17.5 ml TB and grown to mid-log (∼0.35 A_575nm_), whereupon, cells were diluted into buffer and spread on TB plates that were incubated for 24 hr at 30°C, and onto TB and TBamp50 plates that were incubated at 42°C for 24 hr. Survival to Replicative Killing was assessed by dividing the average cfu/ml at 42°C incubation (the cell titers on both TB and TBamp50 plates were equivalent) by average titer for cell dilutions incubated at 30°C. Lane ‘f’ is a summary of the plasmid's effect on Replicative Killing of induced Y836 cells, where NONE indicates the cells were killed upon induction, and FULL reflects high cell survival as determined by colony formation at 42°. The values in parentheses show standard error for at least two independent determinations. Lane “g” shows the level of each plasmid present in the cells at 30°C (noninduced), immediately prior to shifting cells to 42°C (see legend, Fig. 4). The duplicate cultures processed at time 0 were extracted for DNA using Qiagen DNAeasy Kit, estimating 1.0×10^8^ cells per 0.1 A_575nm_ and calculating the amount of cell culture needed for 2.0×10^9^ cells per DNA preparation. All DNA samples were prepared in duplicate. The gel purified bands for the plasmid DNA present in the 0 time cultures was assessed by hybridization as described in Fig. 4.

The spacing interval between the *t_O_-oop-p_O_* sequence and *ori*λ in p50 was modified by deletion or insertion (Fig. S3D) to learn if the spatial orientation between these two regions was important for IP. All the modified versions of p50, *i.e.*, p51, p51kan, and p52, retained IP (Fig. S3C–D). We asked if transcription of *oop* from *p_O_* participated in IP by inactivating the -10 region of *p_O_*, replacing the sequence ATTAT with GCGCG in p27R to stringently assess a requirement for *oop* expression from a high copy *ori*λ plasmid. The resulting plasmid, p27R*p_O_* ^−^ (Fig. 3C), no longer expressed *oop*, as determined by the OOP antisense phenotype/*cII* inactivation assay (see Materials and Methods) and was defective for IP (Fig. 3D), suggesting that transcription from *p_O_* is essential for IP. To distinguish whether the transcription of the downstream *oop* sequence, or just transcription initiation from the *p_O_* promoter was required for IP, the coding sequence of *oop* was modified in plasmid p27R-R45OOP (Fig. 3C). Nucleotides 2–46 of *oop* were replaced with a randomly chosen sequence, edited to remove internal secondary structure formation. For maintaining the self-terminating stem-loop structure of *t_O_*, the distal 31 nucleotides of *oop* were retained, as was the first base pair of the *oop* sequence, corresponding to 5′ pppG of OOP RNA. p27R-R45OOP was unable to serve as an antisense RNA to inactivate *cII* and it was defective for IP (Fig. 3D, columns 1–3). The results with plasmids p27R*p_O_* ^−^ and p27R-R45OOP suggest that transcription of the intact *oop* sequence is required for IP, rather than just transcription initiation from *p_O_*.

The *ori*λ sequence comprises bases 39034–39160 within gene *O* (Fig. 3B), with four 18 bp iteron (ITN1–4) sequences joined to a 38 bp high AT-rich sequence. The binding of O protein to *ori*λ is required for theta-mode replication initiation [15], [19]–[27]. A requirement for the ITN's and AT-rich region for IP was investigated using plasmids p27RΔITN1–4, p27RΔITN3–4, and p27RΔAT (Fig. 3C). The deletion of ITN1–4 or ITN3–4 nullified IP; whereas, the deletion of the AT-rich region was without influence on IP (Fig. 3D, columns a–c). In the *cII* inactivation assay for measuring synthesis of OOP RNA, clear plaques by λ*cI*857(*18*,*12*)P22 were formed on 594 cells transformed with p27RΔITN1–4, p27TΔITN3–4, or p27RΔAT, indicating that each synthesized OOP RNA. Thus, transcription of the *oop* sequence from *p_O_* and the presence of ITN's (particularly ITN3–4) are requirements for IP directed to *rep*λ phage.

### IP silences λ replication initiation

**Figure 4:**
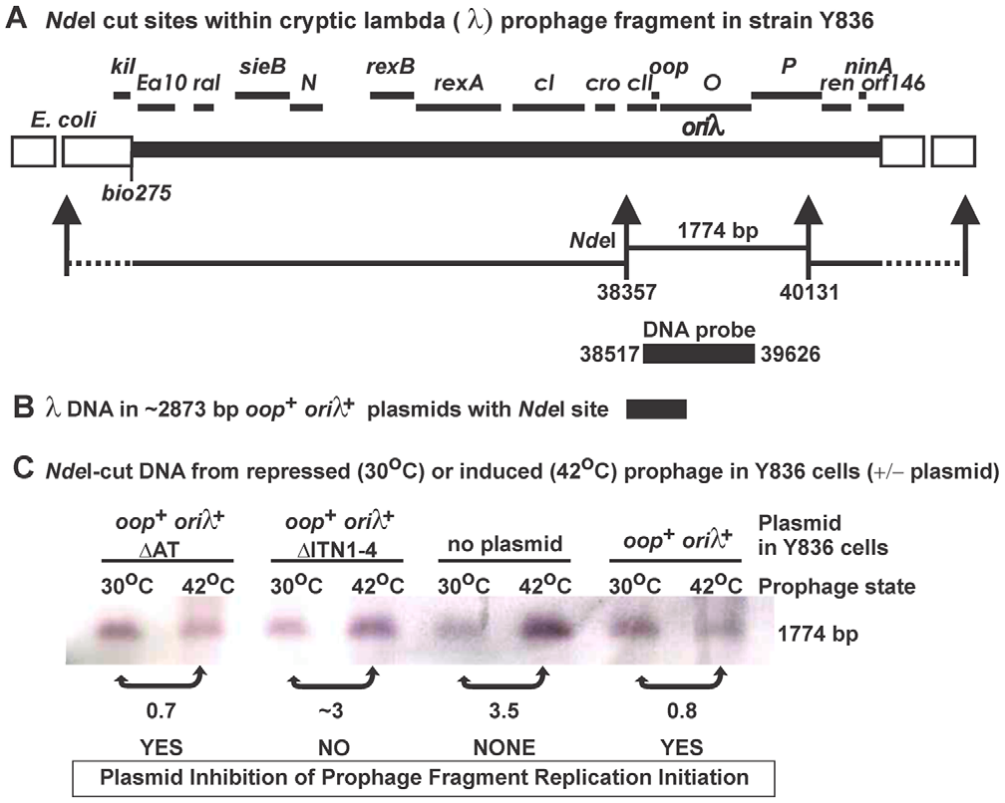
Assay for prophage replication from *ori*λ. This experiment was undertaken in parallel with the experiment shown in columns “e–g” of Fig. 3D. A. Map of λ fragment within Y836 cells. The thick solid line shows λ fragment within the *E. coli* chromosome (open boxes); the *NdeI* restriction sites within λ and chromosome are shown along with the DNA bands formed after cleavage; the λ region amplified to prepare a DNA probe is drawn. B. Map region of the λ DNA fragment cloned within plasmid p27R (2873 bp) (without indicating the small mutational changes within similar λ fragments in the other plasmids). C. Assay for replication initiation from *ori*λ after shifting Y836 culture cells from 30° to 42° to induce transcription and *ori*λ replication from the cryptic prophage. Cultures were grown to mid-log and aliquots were removed at time 0 as described in legend, Fig. 3. Thereupon, cultures were transiently swirled in a 60°C water bath and transferred to a 42°C shaking bath for one hour and aliquots were removed. Cell concentration of the 42°C aliquots was based upon the calculations for 30°C 0-time cultures, and DNA was prepared using Qiagen DNAeasy Kit from 2.0×10^9^ cells. The concentration of extracted DNA was determined by spectrophotometer (A_260nm_ x DNA dilution X 50 ng/ml). The Y836 cellular DNA (2.5 ug of ethanol precipitated and resuspended DNA) was digested 2 hrs with *Nde*I and digests were run on horizontal 0.7% agarose gel, followed by Southern transfer of DNA bands. The Southern blot bands for the 1774 bp chromosomal prophage fragments were each scanned 3X using GE Healthcare software program ImageQuant version 5.2 and the region under the peaks was integrated and averaged. The numbers below the bands compare the relative levels of 1774 bp fragment obtained for induced / noninduced sample *pairs*. Refer to Hayes *et* *al.* [18] for detailed hybridization methodology, and for comparing the effect of a *cI* ^+^ repressor expressed from a plasmid on prophage induction, the influence of host recombination defects on replication initiation from *ori*λ from the prophage in Y836 cells, and the inhibition of replication initiation from *ori*λ by host mutations.

Lambda replicates in two stages. The early or bidirectional (theta) mode from *ori*λ starts within two minutes following thermal de-repression of a λ*cI*[Ts]857 prophage [28]. The late or rolling circle (sigma) replication mode forms linear DNA concatemers, the preferred template for packaging λ DNA into phage heads. The sigma mode arises about 15 min after phage infection of cells [29]–[32]. Skalka *et* *al*. [31] stated that replication via the “early mode occurs only once or twice, after which rolling circle (late) replication predominates.” They suggested that a direct, internal control gene for the turn-off of early replication either “does not exist”, or “must not be expressed in the absence of replication” because early replication products accumulate (after infection or induction) when concatemer formation is destabilized in λ *gam* mutants, or under *fec* ^−^ conditions (involving both λ *red* and host *recA* mutations). The chromosome in strain Y836 (Table 1; Fig. 4A) has an engineered cryptic λ prophage deleted for recombination genes *int-xis-exo-bet-gam-kil* involved in general and site specific recombination [13] and for genes *orf146* ( = *orf*) – *J*b2, including genes required for cell lysis and phage morphogenesis [33], but it encodes the *imm*λ and *rep*λ regions. Transcription of *O*–*P* from *p_R_* is prevented at 30° by the *cI*[Ts]857 encoded temperature sensitive repressor. Inactivating the CI repressor, by shifting cells grown at 30° to 42°, triggers *ori*λ-dependent bi-directional replication initiation from the trapped λ fragment. Initiated replication forks escape leftward and rightward beyond the λ fragment and into the *E. coli* chromosome. This event is lethal to the cell and was termed Replicative-Killing [7], i.e., RK^+^ phenotype [18], [34]. Survivor cells that escape Replicative-Killing (RK^−^ mutants) arise within the RK^+^ starting cells and were found to possess mutations that prevented replication initiation from *ori*λ [13], [14], [33]–[35]. Transducing a *dnaB* mutation (GrpD55) that prevents λ replication initiation (but not *E. coli* DNA synthesis) into the RK^+^ Y836 cells can fully suppress Replicative-Killing without interfering with gene expression from the induced λ fragment [18]. We examined whether plasmids exhibiting the IP phenotype could suppress Replicative-Killing (Fig. 3D, rightward columns e-g). The viability of RK^+^ Y836 cells shifted from 30° to 42° was <0.00001. Similar results were seen when Y836 was transformed with p27R-R45OOP, p27RΔITN1–4, or to a lesser extent with p27R*p_O_* ^−^, indicating that these three plasmids do not suppress the RK^+^ phenotype. Cells transformed with plasmids p27R and p27RΔAT suppressed Replicative-Killing at 42°, suggesting that they interfered with (silenced) theta-mode replication initiation from the chromosomal λ fragment.

We examined if the IP-plasmids could block replication initiation from a thermally induced cI[Ts]857 λ fragment within the Y836 chromosome. Replication initiation arising from the *ori*λ region of the induced cryptic prophage was assessed by probing for a 1774 bp *Nde*I fragment (Fig. 4A–C) following *Nde* I digestion of the Y836 cell chromosome. The probe to the *Nde*I fragment overlapped with each of the λ fragments in the plasmids introduced into Y836, permitting an internal measure of plasmid copy increase. Theta-mode replication initiation increased by about 3-fold from *ori*λ when Y836 cells without a plasmid were shifted from 30° to 42° (Fig. 4C). The *oop* ^+^ *ori*λ^+^ plasmid p27R fully inhibited theta mode replication initiation, in full agreement with the data showing that this plasmid blocked Replicative-killing (Fig. 3C). Cells with p27RΔITN1–4, with a deletion of the four iterons (but not the AT-rich region) was not inhibitory; whereas, the converse plasmid p27RΔAT, modified to remove the high AT-rich sequence but containing ITN1–4, was fully inhibitory to theta-mode replication initiation from the prophage *ori*λ site. The intensity of the replication increase was not as robust as previously seen (Fig. 2 in [18]) where the probe was larger and could detect two λ prophage restriction fragments (i.e., 3675 bp *ori*λ band, and a 4250 bp band showing escape replication), possibly because of the high level of competition for the probe by the λ DNA within the plasmids. Two of the 1774 bp bands at 42°C for cells where *ori*λ replication initiation was inhibited decreased slightly compared to their 30°C counterparts. This may represent some level of DNA extraction variation, or it could be real and represent fragment destruction resulting from abortive *ori*λ replication initiation from the prophage in these strains.

### Escape from IP

We previously showed [18] that marker rescue for *imm*λ recombinants was below the level of detection for Y836 *dnaB*-GrpD55 host cells infected with *imm*434 phage deleted for λ genome regions NinL (*int-red-gam* recombination functions) and NinR (*ren-ninA-ninI*, including Orf and Rap) (Table 1 in [18]). The same result was seen for Y836 *recA* host cells infected with *imm*434 versions of NinR^+^ ΔNinL and ΔNinR ΔNinL phages (Table 2, lines 2–3 in [18]). The GrpD55 locus was suggested linked to *dnaB* [36], and Horbay [37] subsequently determined by sequence analysis that it represented two missense mutations within *dnaB*. The *dnaB-*GrpD55 mutation confers a temperature sensitive phenotype for λ plating but does not prevent *E. coli* replication, cell growth [36]. The EOP of λ on strain W3350 *dnaB*-GrpD55 was significantly reduced compared to W3350 (EOP set  = 1.0). The respective EOP's at 30°, 40° or 42° on the *dnaB*-GrpD55 host were 0.08, 0.01, <0.0001 (for λ*cI*857); 0.2, 0.002, <0.0001 (for λ*imm*434*cI*); and 0.4, 0.04, <0.0001(for *λ*imm434ΔNinR), showing increasing temperature sensitivity for λ replication, while the *E. coli dnaB*-GrpD55 host was able to form effective cell lawns at the elevated temperatures. We define “free-loader” coefficient, as a measure of phage progeny for infections at multiplicity of infection (MOI) 5, per the phage progeny from infections at MOI 0.01 (see Discussion). The availability of λ recombination functions within an infected cell can influence the free-loader coefficient. W3350 *dnaB*-GrpD55 *recA* ^+^ cells were infected at MOI's of 5 or 0.01 with λ deleted for the NinL, NinR, or both recombination regions, then incubated for 90 minutes at 42° and plated for phage burst. Infections with phages λ*imm*434NinL^+^NinR^+^, λ*imm*434NinR^+^ΔNinL, λ*imm*434ΔNinR NinL^+^, and λ*imm*434 ΔNinLΔNinR yielded respective coefficients of 1065 (+/−18 std. error), 502 (+/− 31), 136 (+/−10), and 111 (+/− 27), suggesting that the λ NinR and NinL recombination functions can influence phage burst from multiply infected cells where the infecting phages are blocked for theta-mode *ori*λ-dependent replication initiation by the *dnaB*-GrpD55 mutation. This result supports our prior suggestion [18] that *ori*-specific theta-mode replication initiation, dependent upon P-DnaB interaction, can be bypassed in multiply infected cells, *i.e.,* phage replication can likely be driven by intermediates derived via homologous recombination between co-infecting phage genomes.

The results from Fig's 1, 2, 3 and 4 suggest that IP serves to block / silence replication initiation from *ori*λ. We examined whether IP could be bypassed, comparing the bursts from singly infected (low MOI, 0.01), or multiply infected (high MOI, 5) cells (Table 5). Infections of wild type host strains W3350 and 594 at either MOI's of 5 or 0.01 with *rep*λ or *rep*P22 phages produced essentially equivalent bursts. A similar result was seen for *rep*P22 phage infections of W3350 *dnaB*-GrpD55 cells at either MOI 5 or 0.1. There was essentially no burst (background level) when the *rep*λ phage infected W3350 *dnaB*-GrpD55 cells at an MOI of 0.01; however, the phage burst was equivalent to that on the W3350 cells when the W3350 *dnaB*-GrpD55 cells were multiply infected at an MOI of 5. Thus, while the altered DnaB protein [GrpD55 allele] interferes with the P-DnaB interaction required for theta-mode λ replication initiation, it can still apparently drive λ or *E. coli* DNA synthesis that is independent of P. Placing multiple copies of a recombination proficient λ genome within a cell appears to bypass the P-DnaB interaction at *ori*λ required for the theta-mode of λ replication initiation. Similarly, 594 cells with plasmid p27R (*oop* ^+^ *ori*λ^+^) prevented phage burst from cells infected at MOI 0.01. But when these same cells were infected at MOI 5, IP was suppressed (bypassed). 594 cells with p27R*p_O_* ^−^, which is defective for IP, yielded an essentially similar *rep*λ phage burst at MOI 0.01 as when 594 cells without the plasmid were infected. These results suggest that IP serves to silence / inhibit theta-mode *ori*λ replication initiation and that multiple copies of recombination-proficient λ genomes can, at some level, bypass this essential requirement for replication initiation from a single prophage or from one infecting λ genome.

### Suppression of Inhibition Phenotype (Sip) by λ mutants and hybrids

**Figure 5:**
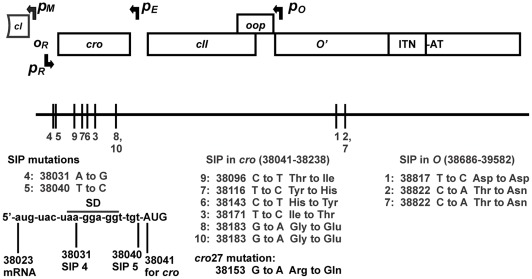
Sequences of Sip and *cro*27 mutations. For an alternative interpretation of the effect of Sip mutations on gene expression from *p_E_* refer to Fig. S7B. GeneBank Accession #'s for Sip mutants: 1 (DQ372057.1), 2 (DQ372058.1), 3 (DQ372059.1), 4 (DQ372060). Newer data for all Sip phages and for *cro*27 mutation in λ*cI*857*cro*27 was submitted, BankIt1376628 : (12). Phage λ*cI*[Ts]857*cro*27 was found to be WT between the end of *cII* and start of *P*, i.e., *O* ^+^.

We looked for a target of IP by i) characterizing 10 independent (Sip) mutants of λ*cI*857 (Fig. 5); and ii) by screening for IP-escape, testing λ mutants and hybrid phages (Fig.'s S4, [Supplementary-material pone.0036498.s005]S5). We first asked if insertion by homologous recombination (of the Amp^R^ *oop* ^+^ *ori*λ^+^ plasmid into the infecting phage) was responsible for Sip (Fig. S6 and Supplemental Methods S1), and eliminated this possibility. The *cI* – *P* regions were sequenced for 10 independent Sip phage isolates, and for λ*cI*857*cro27* with a null mutation in *cro*, Fig. 5 [11], [28], [38]–[40]. Three sip mutations, Sip 1, 2, 7 arose at two sites in *O* to the left of the ITN sequences, of which mutations Sip 2 and 7 introduced different changes in the same codon by altering position 38822. Five other Sip mutations (3, 6, 7, 8, 9, and 10) introduced missense changes within *cro*. Another Sip mutation (Sip 4) altered the ribosomal binding (SD) site for *cro* and another (Sip 5) changed the base preceding the AUG for *cro*. One of the sip phage (Sip7) was mutated in both *cro* and *O*. By conventional logic, the Sip mutations in *cro* might function by reducing Cro down regulation of *p_R_* and thus increase *O* expression, or the Sip mutations in *O* increase *O* expression or activity.

Alternatively, several of the Sip mutants conferred missense mutations in an 81 codon open reading frame, PreX; these included five Sip mutations (of which Sip6 eliminated the PreX start codon); plus the “se” mutations (described above) introduce missense changes into PreX (Fig. S7). PreX can only be expressed via high level establishment mode *p_E_-preX-cI-rexA-rexB* mRNA synthesis (i.e., 20–100X level of *pM-cI* transcription [28], [39], [40]), requiring CII activation at *p_E_* [3]. The *p_E_-cI* transcript is antisense to *cro*, and the possible PreX reading frame from it would overlap 13 codons at the N-terminal end of *cI*, all of *oR*/*pR* region, and 35 codons of *cro*, and would be expressed from the same reading frame as *cro*, but the opposite coding strand (Fig. S7).

Since six of the λ*cI*857-derived Sip mutants produced five missense changes in *cro* (two independent Sip mutations, 8 and 10, each changed base pair 38183 in *cro*), we examined if any Sip mutants exhibited the λ*cI*857*cro*27 plating phenotype. Phage λ*cI*857*cro*27 has the interesting property of forming plaques at 37–39°, but not at 30° or 42° [38], [40]–[42], and of exhibiting a phenotype within infected cells termed Cro lethality (See [40] for a discussion of *Cro* lethality concept relative to *rexA-rexB* expression, translational frameshift sites within [43], and possible effect upon [14] high levels of *p_E_-preX-cI-rexA-rexB* expression (Fig. S7A,B) from an induced *cro*-defective λ lysogen or infecting phage.) Our isolate of λ*cI*857*cro*27 carried a single G-A transition (Arg to Gln) at base 38153 in *cro* (Fig. 5), nullifying *cro* activity. Only the Sip7 phage shared a nearly similar plating phenotype with λ*cI*857*cro*27 by forming faint plaques at an EOP of <10^−3^ at 30°, tiny-faint plaques at EOP 0.3 at 42°, and 1 mm clear plaques at 37 and 39°. Sip phages 1–6 and 8–10 formed 0.5–1.0 mm turbid plaques on 594 host cells at 30°, and about 1 mm clear plaques at 37°. Only the Sip 4 and 8 phages plated with slightly reduced EOP, i.e., by 3 or 13-fold, at 30° compared to 37°. Alternatively, we asked if λ*cI*857*cro*27 can escape IP, i.e., if it shares properties with the λ*cI*857Sip phages, and found that the *cro*27 allele did not confer a Sip phenotype (Table S1). Thus, simply inactivating Cro does not directly confer a Sip phenotype, and so the Sip mutations must have another effect.

The inability of the *rep*λ phage λ*cI*857 to escape IP was not modulated by the CI repressor, reflected by equally IP-sensitive *rep*λ phages λwt (*cI* ^+^), and phenotypically CI^−^ (lysogenization-defective) phages: λ*cI*72 (*cI* ^–^), and by phages with CI-defective phenotype that escape replicative inhibition, i.e., λ*oR*/*pR* point mutations (λse mutants: 100a, 101b, and 109b (Table 1, Fig. S7C), and λ*cI*90c17 (Table 1), where *pR*-independent transcription [44], [45] arises via the c17 insertion downstream from *pR*). The *rep*λ phages λvir, λ*imm*21*cI* and λ*imm*434*cI* partially escaped IP, plating with EOP's of 0.1 or higher (Fig. S4A), but their plaque sizes were reduced. The sequence of λ*imm*434*cI* was identical to λ throughout the *cII-O* interval (Fig. S5). λvir is mutated in both *o_R_2* and *o_R_1* at bases 37979 and 38007 [2], [34], respectively, although, it is unclear what other mutations it possesses. The λ*imm*21 hybrid had base alterations within the *cII-oop* overlap (Fig. S5) and a silent TGC to TGT codon change at 39,033 (not shown), one base left of the ITN1 sequence in *O*.

**Table 5:** *ori*λ-dependent DNA replication inhibition is bypassed in multiply infected cells.

Host Strain	Burst of infecting phage per cell at indicated MOI ^a^			
	λ*cI*857 [*rep*λ]		λ*cI*857(*18,12*)P22 b [*rep*P22]	
	MOI 5	MOI 0.01	MOI 5	MOI 0.01
W3350	35.5+/−5.3	31.0+/−6.2	13.8+/−1.4	11.3+/−3.4
W3350 *dnaB*grpD55	31.6+/−16.3	1.34+/−0.7	19.2+/−7.3	17.1+/−3.2
594	25.8+/−4.2	25.3+/−8.5	14.6+/−3.8	9.4+/−0.8
594[pBR322]	22.4+/−4.8	26.0+/−12.3	9.0+/−1.0	5.7+/−0.1
594[*oop* ^+^ *ori*λ^+^]	21.1+/−8.2	1.1+/−0.7	6.8+/−0.6	6.1+/−0.7
594[*oop* ^+^ *p_O_*− *ori*λ^+^]	27.3+/−7.2	19.8+/−6.6	10.9+/−0.4	6.9+/−2.2

a Burst at 110 min after infecting cells. The results are expressed as phage burst (# phage particles released per infective center) +/− standard error. Each value represents the average of ≥2 separate trials.

b See [78] for host requirements for growth of λ-P22 hybrid.

Plaque size is a qualitative measure of phage development or burst, and we previously found that impeding λ replication significantly reduced normal plaque size [18]. Thorough examination revealed that the plaques formed by λ*imm*434*cI* on 594[*oop* ^+^ *ori*λ^+^] cells were barely visible, i.e., 5% of their normal diameter on 594 host cells (Fig.S4C) and λ*imm*21*cI* plaques were 35% their normal diameter. Plaques formed on 594[*ori*λ^+^] cells by the *rep*λ phages (Fig. S4C) were reduced in plaque diameter by about half, in agreement with the observations that *ori*λ^+^ plasmids partially interfere with phage maturation.

To help ascertain why the *rep*λ phages λ*imm*434*cI*, and to a greater extent λ*imm*21*cI*, partially escaped IP, their *oop-rep* regions were sequenced (Fig. S5). While phage 434 has three base changes within the *oop* sequence, the λ*imm*434*cI* hybrid sequence was equivalent to λ. The λ*imm*21*cI* hybrid shared the same sequence as phage 21, with an expected altered sequence within *cII* left of *oop*, and differences within the *oop / cII* overlap region (Fig.'s S1, [Supplementary-material pone.0036498.s005]S5). The λ/P22 hybrid, i.e., λ*cI*[Ts]857(*18,12*)P22 that was insensitive to IP, carried the λ version of *cII*, yet differed: by one base (37673) within *oop*, by one base (36689) just right of the common -10 sequence (ATTAGG) for the *oop* promoter *p_O_*, and completely diverged rightward from the λ sequence at base -19 (38694) within *p_O_*, so that the -35 region's for the *p_O_* promoters for λ and for λ/P22 hybrid were distinct (Fig.S5) as were downstream λ genes *O -P* [2] and P22 genes *orf*48-*18–12* [46] (Fig. S2).

All of the *rep*λ phages formed plaques with ∼120% larger diameters on 594[*oop* ^+^] vs. 594 cells (Fig. S4C), suggesting that OOP RNA can stimulate *rep*λ lytic growth. The C-terminal 55 nt including the stop codon for gene *cII* overlap the 3′-end of *oop* (Fig. S1). The last 17 amino acids of *cII* are not required for CII activity, but this region is necessary for CII regulation by OOP [5]. The infection of *cII* ^+^-λ phages into cells with plasmids expressing OOP micro RNA, which is antisense to *cII* [47] (Fig. 2), creates a *cII-*defective phenotype [48] resulting in clear plaques at 30° even for the hybrid λ*cI*857(*18,12*)P22. Even our *cI* ^+^ version of λ*imm*21 gave turbid plaques on 594, but clear on 594[*oop* ^+^] host cells, suggesting that the five base changes within the *oop / cII* overlap region do not prevent OOP RNA (made from *oop* ^+^ plasmid) from serving as an antisense RNA to *cII* expression from *imm*21. Clearly, infecting *cII* ^+^ phages into cells expressing OOP RNA creates a phenotypic *cII*-defective condition, characterized by no *p_M_-preX-cI-rexA-rexB* transcription, no *cro* antisense RNA, and lytic phage growth. Thus, we did not consider it relevant to evaluate independent missense *cII* ^-^ phages, all of which map left of the *cII/oop* overlap [3]. In hundreds of *cro* ^+^ *cII* ^+^ prophage induction experiments, for example [4], [28], [39], [40], [49], no *l*-strand transcription attributable to *p_E_* was ever detected (Hayes lab results). This result, coupled which with our current understanding of the role of OOP as an antisense regulator of *cII* expression, suggests that the synthesis of OOP RNA under the conditions described herein will prevent *p_E_* transcription from infecting phage or induced prophage. But, an OOP block to *p_E_* transcription is insufficient on its own to explain CI-independent IP, i.e., *oop* ^+^ Δ*ori*λ plasmids were defective in IP.

We examined the IP-sensitivity of a phage deleted for *cII-oop.* The interval between AUG for *cII* and second codon for *O* in phage λ*c*I^+^Δ*cII* ( = Δ*oop*) [50] was deleted (i.e., λ bp 38363–38688; we confirmed by sequencing two isolates). The deletion fused the retained -35 region of the *oop* promoter, *p_O_* (leftward from bp –14 at 38689), with the sequence left of the second codon for *cII* (bp 38362), changing the -10 region for *p_O_* from ATTATG to CATATG, which might still support *p_O_*-dependent leftward transcription. The λ*c*I^+^Δ*cII* phage partially escaped IP, forming pinprick-ghost plaques (impractical to quantitate/measure) on 594[*oop* ^+^ *-ori*λ^+^], considerably smaller than those of λ*imm*434*cI* on the same host (Fig. S4C). The λ*c*I^+^Δ*cII* phage was much more sensitive to copies of *ori*λ and formed very much smaller plaques than λ*imm*434*cI* or λ*cI*857 phages on 594[*ori*λ^+^] and 594[*oop p_O_* **^−^** *-ori*λ^+^] cell lawns; yet it was capable of forming large clear plaques at EOP of 1 on 594 and 594[*oop* ^+^] cells. Further analysis is needed to explain the paradox that *rep*λ phages retaining the *cII-oop* region are sensitive to IP (requiring OOP and *ori*λ) yet their development is not curtailed by the presence of competing *ori*λ plasmids; whereas, deleting *cII-oop* has the opposite effect.

## Discussion

### Replicative inhibition

We previously showed that the hybrid phage λ*cI*857(*18*,*12*)P22, with the *rep*λ region swapped by *rep*P22, was extremely sensitive to CI-dependent replicative inhibition, and by comparison, λ*cI*72, the λ se mutants, and λ*cI*90c17 were respectively 4.6, 27–76, and 173 fold less sensitive [13]. This result illustrates that CI-dependent replicative inhibition does not directly target the *rep* region, but rather, transcriptional activation of *rep*. In contrast, the *rep*P22 phage escaped CI-independent replicative inhibition; whereas, the *rep*λ phages as λ*cI*72, the λ se mutants, and λ*cI*90 c17 were fully sensitive. Therefore, we would assert that the CI-dependent (blocking transcriptional activation of the *rep* region) and the CI-independent (IP directed theta mode replication silencing) forms of replicative inhibition are completely distinct, and that their mechanisms are likely different, even if they share the same end result.

### Requirement for IP

We have provided additional understanding of the observation, termed here IP (Inhibition Phenotype), whereby host cells with plasmids containing the *oop-ori*λ region of the lambda genome inhibited phage plating. This region includes several *cis-*acting target sites, for example, the iteron sequences, ITN1–4, bound by *O* protein and sites for promoter, *p_O_*, and terminator, *t_O_*, for the 77nt OOP micro RNA (Fig's S1,2,3B,5)[51]. In summary: *i*) Plasmids containing the λ *t_O_-oop-p_O_* through *ori*λ DNA sequence inhibited the development of *rep*λ infecting, or an induced λ*cI*857 prophage, and neither the *oop* nor *ori*λ regions, separately, could account for IP. *ii*) IP was independent of the activity of λ repressors CI and Cro, *iii*) A λ/P22 hybrid with *rep*P22 was insensitive to plasmids containing the *t_O_-oop-p_O_*λ and *ori*λ DNA sequences, suggesting that IP is directed to a *rep*λ function. *iv*) Sequence analysis revealed that the λ/P22 hybrid contained *imm*λ, an essentially intact (one base change) *oop* sequence, a hybrid *p_O_* promoter with a λ -10 region and P22 –35 region, and the substitution of λ genes *O-P* with P22 genes *orf48*–*18*– *12* [37], [51]. *v*) OOP RNA synthesis from the *oop* ^+^ plasmids channeled both the λ/P22 and λ*imm*21 phages into a lytic mode to form clear plaques, suggesting the level of OOP RNA made was sufficient to serve as an antisense regulator of *cII* expression from the *p_R_* transcript(s). [5], [47]. *vi*) A dissection of the contributions to IP revealed that an *oop* ^+^ plasmid deleted for the AT rich region of *ori*λ was fully functional for IP, *oop* ^+^ plasmids deleted for ITN1–4 or ITN3–4 were defective for IP, and *ori*λ^+^-containing plasmids substituted for 45bp within *oop*, or inactivating the *p_O_* promoter for *oop* transcription, were defective for IP.

### Phage escape from IP

In summary: 1) Two types of full escape from *oop* ^+^ *ori*λ^+^ plasmid-dependent-IP were observed: *i*) substitution of *O-P* in λ by *orf*48*-18*−*12* in the λ/P22 hybrid (Fig. S5) enabled the hybrid to escape IP, even though its *cII* expression was inactivated by OOP RNA; and *ii*) Sip mutations within or near *cro* or in *O* suppressed IP. 2) Some *rep*λ phage partially escaped *oop* ^+^ *ori*λ^+^ plasmid-dependent IP, but phage development was retarded (as evidenced by reduced EOP and plaque size). 3) Phages that could escape CI-dependent replicative inhibition were unable to suppress IP. This result refutes a hypothesis that natural or mutational events that increase transcription from *p_R_*, e.g., by limiting Cro or CI binding to *o_R_,* or introducing downstream promoters, will augment transcriptional activation of *ori*λ, and in turn promote theta-mode-*ori*λ-dependent replication initiation, and suppress IP. Another explanation is needed. Anderl and Klein [52] suggested that if the ratio of DNA:O protein is increased, theta-mode replication initiation will be inhibited due to titration of O protein, which suggests that plasmid-borne *ori*λ iteron sites could act as competitor origins, sequestering the O protein made by infecting *rep*λ phages. The “handcuffing” analogy for dimer formation [25] between O proteins binding to the iteron sequences in several *ori*λ sites could serve as a model for blocking the formation / completion / processing of a preprimosomal complex. The minimum molar ratio [53] of O protein:*ori*λ (termed O-some [54] complex) that was required for strand unwinding was 20∶1. When additional *ori*λ regions are present, or if multiple interacting O-*ori*λ complexes are formed, it is unlikely that this molar ratio will be achieved. Our results suggest that handcuffing cannot account for IP, even if multiple *ori*λ targets bind excess O protein. Cells with multiple copies of two plasmids lacking *oop* sequence, but encoding an intact gene *O/ori*λ, did not reduce EOP, i.e., exhibit IP, whether or not *O* was expressed.

### Theta-mode replication silencing by IP

The loading of DnaB onto ssDNA, formed by strand separation within the high-AT-rich region of *ori*λ, was suggested to mark the end of the initiation phase of λ theta mode DNA replication [55]. Previously, we confirmed that theta-mode *ori*λ-dependent prophage replication initiation, which requires P interaction with, and loading of, DnaB, was inhibited if the host carried the *dnaB*-GrpD55 mutation, yet there was no obvious influence of this allele on *E. coli* DNA propagation [18]. Herein, we observed that both theta-mode replication from *ori*λ, and its manifestation, i.e., the Replicative Killing of induced cells (dependent upon triggering theta-mode replication from a trapped, defective λ prophage) was prevented in cells with plasmids exhibiting IP. Both observations strongly suggest that theta-mode replication initiation is silenced, *in trans*, by the *oop* ^+^ *ori*λ^+^ plasmids. Blocks to theta-mode replication initiation from an infecting phage, by cellular *oop* ^+^ *ori*λ^+^ plasmid copies or by the chromosomal *dnaB*-GrpD55 mutation, could be bypassed by multiply infecting such cells with λ. This result is not without precedent. Freifelder *et* *al.* [56] infected nonpermissive cells at MOI's between 0.01 and 40 with λ*cI*857*P*am3 phages that were variously inactivated for integration or Red recombination functions. For their Int^+^ Red^+^ variant, they showed an increase in phage burst of 240-fold between MOI's of 0.01 (transmission coefficient 0.001) and 10 (transmission coefficient of 0.24), yet the λ*cI*857*P*am3 phage was unable to form plaques on nonpermissive cells; and in our hands the *P*am3 mutation reverts at a frequency of <10^−7^. Freifelder *et* *al.* [56] concluded that if recombination is reduced, the ability to produce mature phage was markedly reduced. McMillin and Russo [57] reported that under conditions which block λ DNA duplication, unduplicated λ can mature, including molecules which have recombined in the host. Stahl *et* *al.* [58] extended this observation, coining the term “free-loader” phage to describe phage produced under replication-blocked conditions, whose synthesis depended upon bacterial and phage recombination systems. We borrowed this concept, using “free-loader coefficient” to describe the influence of phage recombination functions on λ progeny from infected *dnaB*-GrpD55 cells in which the infecting phage genome cannot initiate theta-mode replication. We showed that phage recombination functions from both NinL and NinR regions can influence by up to ten-fold the phage progeny released from multiply infected *dnaB*-G*rpD55* host cells, supporting the Freifelder *et* *al*. [56] conclusion. Sclafani and Wechsler [59] showed that at low MOI, no λ particles were produced in cells lacking a functional *dnaB* product; yet at high MOI, a significant proportion of the cells can produce phage. Thus, the bypass of an *ori*λ replication block in multiply infected cells could depend upon a recombination-driven replication shunt, possibly analogous to the replisome invasion mechanism described by Poteete [60]. It is recognized that if a cell contains ≥2 circularized λ genomes, recombination between the monomers can produce an invading strand which could lead to rolling circle replication, independent of *ori*λ [61]. Presumably, recombination / replication intermediates can be formed that produce packageable, concatemeric DNA by the introduction of a nick into one of the DNA strands of a λ monomer, enabling rolling circle replication initiating from the 3′-OH end of the nick, or by recombination between homologous λ DNA segments. It was proposed that double-strand break repair recombination intermediates in *E. coli* are capable of initiating and undergoing DNA replication [62], [63]. It is possible that the circularized λ genomes produce linear multimers, formed by the rolling circle type of plasmid replication dependent on the RecF recombination pathway [64]–[67].

The potential to bypass theta-mode replication initiation via recombination suggests that there is no obligatory order / mechanism for triggering late mode λ replication from the early *ori*λ-dependent replication products. Alternatively, the extensive evidence for a shift from early to a late replication mode supports the possibility that some natural mechanism can inhibit early theta-mode replication initiation. Two events come to mind where theta-mode replication initiation is undesired and would best be silenced. Theta-mode bidirectional replication forks arising from a λ DNA copy that is integrating, or has integrated, into the host chromosome will kill the potential lysogen via the escape replication (Replicative Killing). The initiation of theta replication from linear concatemeric DNA might inhibit genomic DNA packaging into the phage head. Our results for plasmid based IP suggest that there is a natural mechanism for silencing theta-mode replication initiation, *i.e.* the buildup of λ genomes with *oop* ^+^ *ori*λ^+^ sequence.

### Toward a mechanism for IP

There are a number of ways *oop* expression could influence transcriptional activation of *ori*λ: *i*) OOP antisense RNA binding the *p_R_* transcript could promote degradation of the downstream *cII-O-P* transcript, in turn limiting transcriptional activation of *ori*λ and *O-P* expression. *ii*) Cells expressing OOP antisense RNA can nullify CII formation, eliminating *p_E_-preX-cI-rexA-rexB* transcription and the (little appreciated) potential of this mRNA to permit a) high CI repressor buildup, b) hypothetical *orf-*preX expression, or c) high level *p_E_*-promoted antisense RNA to *cro* expression, in turn, reducing Cro buildup and interference with transcription from *p_R_* (Fig. S7). Since the *rep*P22 phage λ*cI*857(*18,12*)P22 was insensitive to IP, yet almost fully shared the same *cI-p_R_-cro-cII-oop* sequence as *rep*λ phages, it seems unlikely that the contribution of *oop* to CI-independent IP simply involves OOP serving as an antisense RNA to the *p_R_-cII* mRNA, or events that increase transcription from *p_R_*, but they might explain why cells with an *oop* ^+^ plasmid can stimulate phage maturation (i.e., support larger plaques). Overall, the results suggest that OOP RNA expression from an *oop-ori*λ DNA template increases the sensitivity of *rep*λ genomes to competing *ori*λ sequences, with the outcome of silencing theta mode replication initiation from the *ori*λ sites. This is a new idea in search of an explanation. Some form of molecular coupling between *oop* expression and *ori*λ may serve to block the formation or completion of the preprimosomal complex. Several old observations remain a mystery regarding the regulation of *oop* expression. A low level of *p_O_* transcription arises from a repressed prophage [4], which, if extrapolated would additively increase the level of OOP in cells with multiple *oop* ^+^ plasmids. This low level transcript was discovered because its expression increased about 40-fold between 5 to 12 minutes following the thermal induction of a cryptic λ prophage (as in Fig. 4) [4], [28]. The increase was linked to phage replication, since a prophage deleted for *P* showed no OOP increase [4], nor was there an increase from intact λ prophages in cells with Ts host *dnaB* or *dnaG* genes, or prophage with *O, P,* or *ori*λ mutations [49] which we have confirmed by sequence analysis. While one might explain this as a gene dosage effect, the level of induced *oop* expression was about the same from an induced defective prophage [49] as from an induced λ*cI*857*Sam7* prophage defective for cell lysis (Table S2), where we typically see between 30 -200^+^ fold increase in phage particles; or when λ was induced in cells with a Ts *dnaE* mutation blocking DNA fork progression [49]. This coupling between replication events at *ori*λ, and *oop* expression, still requires an explanation.

## Materials and Methods

### Reagents and media

Growth experiments were carried out using tryptone broth (TB; 10 g Bacto-tryptone and 5 g NaCl per liter), TB plates (TB with 11 g Bacto-agar per liter) and TB top agar (TB with 6.5 g Bacto-agar per liter). Ampicillin was added to a final concentration of 50 μg/ml where required. Ф80 buffer (0.1 M NaCl, 0.01 M Tris-HCl, pH 7.6) was utilized for cell culture and phage dilutions, TE (0.01 M Na_2_ EDTA, 0.01 M Tris-HCl pH 7.6) and TE* (TE but with 0.001 M Na_2_ EDTA) buffers were used for DNA storage and manipulation of DNA, respectively. TM buffer (0.01 M MgSO_4_, 0.01 M Tris-HCl, pH 7.6) was used in phage burst assays. TBE buffer (0.089 M Boric acid, 0.002 M Na_2_EDTA, 0.089 M Tris-HCl, pH 8) was used to make agarose gels and as running buffer during electrophoresis. Restriction enzymes and T4 DNA ligase were from New England Biolabs. *Taq* DNA polymerase was from Invitrogen and New England Biolabs. Oligonucleotides were from Sigma Aldrich and Integrated DNA Technologies, Inc. Plasmid DNA was isolated using Promega Wizard Plus SV Mini and Midi prep, or Qiagen miniprep kits. DNA was isolated from gels using the Qiagen gel extraction kit, and reaction fragments were purified using the Qiagen QIA quick PCR purification kit.

### Bacteria, bacteriophage, and plasmids

Table 1 shows the *E. coli* K-12 and bacteriophage strains and Table 2 and Fig.'s 1, 3, S3 show the plasmids employed. All of the plasmids were derived from plasmid pCH1 [11] prepared by ligating the λ34500–41731 *Bam*HI fragment into the unique *Bam*HI site of pBR322. The λ sequences are as described by Daniels *et* *al.* [2]. The λ fragment orientation in pCH1: λ base pair 41731 was closest to the N-terminal end of the interrupted *tet* gene.

### Plaque Assay

*Rep*λ  = λ*cI*857 and *rep*P22  =  λ*cI* 857(*18,12*)P22 infecting phages were plated on several plasmid-containing host cell strains to measure plasmid-mediated inhibition of phage plating. An aliquot (0.25 ml) of a fresh overnight cell culture was mixed with 3 ml of warm TB top agar and 0.1 ml of diluted *rep*λ or *rep*P22 phage lysate, and poured over TB or TB+Amp plates. Plates were incubated at 30° overnight and plaques counted. The results were expressed as EOP, *i.e.* phage titer on 594[test plasmid] / phage titer on plasmid free host 594 cells.

### Prophage Induction Assay

The *rep*λ and *rep*P22 prophages were thermally induced in lysogenic cells transformed with plasmids containing various λ fragments. Lysogenic cells were grown at 30° in 20-ml TB (+/– Amp) in a shaking bath to A575 nm = 0.15. The *cI*[Ts]857 prophage in the cells was synchronously induced by swirling the culture flask in a 55–60°C water bath for 15 seconds and then transferring to a 42° shaking water bath to denature the repressor. The culture absorbance was monitored at 30 minute intervals over five hours. Each culture assay was repeated, the several results were averaged and the standard error determined.

### Phage Burst

Host cells transformed with plasmids containing various λ fragments were infected with a *rep*λ or a *rep*P22 phage at a high or low MOI. The phage particles released per infected cell (*i.e.* phage burst) were measured for each infection. Protocol: 16–18 hour culture cells grown at 30° in TB (+/– Amp) were pelleted and resuspended in an equal volume of Φ80 buffer. A cell aliquot (0.1-ml) was mixed with 0.2-ml of ice cold 0.01 M MgCl_2_/CaCl_2_ plus an appropriate volume of sterile phage lysate needed for MOIs of 5 or 0.01. The cell-phage infection mix was held on ice for 15 min to permit phage attachment and then transferred (time zero for measuring infective centers) to a stationary 42° air incubator for 10 min to permit phage infection. The cell-phage mixture was pelleted and resuspended (2X) in Φ80 buffer and the third cell pellet was resuspended in 0.4 ml pre-warmed 42° TB. Half of the resuspended cells (0.2-ml) were inoculated to 20 ml TB (+/– Amp), incubated with shaking at 42°, and aliquots were removed after 65 and 110 min from the time of inoculation to determine phage titer. The second half (0.2-ml) of the washed cell-phage mixture (first held 15 min on ice and then at 42° for 10 min) was immediately pelleted. The supernatant was used to measure the unattached phage remaining after the attachment and infection steps, and the cell pellet was resuspended, diluted, and aliquots were mixed with sensitive cells, top agar, and overlayed on a TB agar plate. Each plaque that arose on the plate was from a potential infective center (an infected cell that has not yet lysed). The phage burst (number of phage released per number of infective centers) was determined for the 65 and 110 min infections, correcting for the phage particles that did not attach to cells.

### OOP Phenotype/CII Inactivation Assay

The last 17 codons of *cII* are not required for CII activity, but are necessary for CII regulation by OOP [5]. The C-terminal 52 nucleotides plus the stop codon for gene *cII* overlap the 3′-end of *oop*. The expression of OOP antisense RNA from a plasmid prevents lambda CII expression [48], resulting in an otherwise *cII* ^+^ phage producing clear, rather than turbid, plaques. An aliquot (0.3 ml) of stationary phase cells being tested for OOP activity was mixed with 0.1 ml of diluted λ*cI*857(*18,12*)P22 phage plus 3 ml of warm TB top agar and poured onto TB plates. The plates were incubated overnight at 30°. Plaque morphology was then determined as clear (OOP^+^) or turbid (OOP^–^).

### Plasmid Sequence Modification

We supplied primers and DNA template to the service at National Research Council/Plant Biotechnology Institute, Saskatoon to confirm the λ-region sequences for the plasmids employed and to verify the mutations introduced into plasmid p27R. PCR mutagenesis was used to modify the *t_O_-oop-p_O_* and *ori*λ plasmid DNA sequences using the SOEing technique [68]. p27R*p_O_* ^−^ (*t_O_-oop-p_O_* ^–^ *-ori*λ^+^): For mutating the -10 region of the *p_O_* promoter in p27R, two primers were made that contained the sequence 5′GCGCG3′ in place of the wt sequence 5′ATTAT3′ at λ bases 38684–λ38688. One primer contained the *l*-strand sequence λ bases 38671–38700 (LPo3) and the other contained the *r*-strand sequence λ bases 38700–38671 (RPo2) (Table 3). The p27R template was PCR amplified with the mutated primers and with primers LPo1 (5′ *Nde*I site and λ bases 38357–38372) and RPo4 (5′ *EcoR*I site and λ bases 39172–39153) in a two-step PCR technique. Both for this plasmid and for those described below, the final PCR product was digested with *Nde*I and *Eco*RI and ligated into the larger (∼2000 bp Amp + ColEI origin) fragment resulting from p27R *Nde*I and *Eco*RI digestion. p27R-R45OOP: Bases 2–46 of the *oop* gene coding sequence in p27R were mutated. Two primers were made to contain “random” bases (screened to eliminate secondary structures) replacing λ bases 38630–38674 of the wild type *oop* sequence. One primer contained the *l*-strand sequence (LROOP3) and the other contained the *r*-strand sequence (RROOP2) (Table 3). The p27R template was PCR amplified with the mutated primers and with primers LPo1 and RPo4 (Table 3). p27RΔITN1–4: Two hybrid primers were made to delete iterons (ITN) 1–4, each with sequences flanking the iterons. LΔITN1–4 contained the λ bases 39014–39033 fused to 39120–39144, while RΔITN1–4 contained the same sequence on the *r*-strand (Table 3). These two primers, in conjunction with LPo1 and RPo4, were used for deleting λ bases 39044–39119 (*i.e.* 87 nt of ITNS 1–4). p27RΔITN3–4: Two hybrid primers were made for deleting iterons 3 and 4 from p27R. LΔITN3–4 contained λ bases 39058–39077 fused to 39120–39144, while RΔITN3–4 contained the same sequence on the *r*-strand (Table 3). These two primers along with LPo1 and RPo4 were used to delete λ bases 39078–39119 (*i.e.* 41 nt comprising iterons 3 and 4). pHB27RΔAT: Primers LPo1 (5′ *Nde*I site and λ38357–38372) and RΔAT1 (5′ EcoRI site and λ39127–39113) were used to amplify the pHB27R λ DNA fragment. The resulting PCR fragment was digested with *Nde*I and *Eco*RI and cloned into the 2000 bp pBR322 fragment from pHB27R digested with *Nde*I and *Eco*RI. The plasmid pHB27RΔAT was shown to be deleted for λ bases 39,128–39172, removing the AT rich region of *ori*λ (Table 2).

### Isolation and sequencing Sip mutants

λ*cI*857 formed small plaques at a frequency of ≤10^−6^ on 594[*oop-ori*λ] cells. An individual plaque from ten separate isolations was transferred by a sterile toothpick to 10 ul buffer (10mM Tris-HCL, 10 mM MgCl2, pH 7.6) and spread using sterile paper strips onto a fresh agar overlay of these cells. This procedure was repeated (as many as 13 times) yet always produced plaques that were heterogeneous in size on the 594[*oop-ori*λ] cells. Each of the ten independent Sip phages were plated on 594 host cells (without plasmid) and a single plaque was used to prepare a phage lysate. Single plaques arising from these lysates were sequenced from gene *cI* into *P* (λ bases 37905–39191) using primers LMH29 (37905–37922: 5′-CTGCTCTTGTGTTAATGG), L22 (38517–38534: TGCTGCTTGCTGTTCTTG), RPG6 (38569–38552: CAATCGAGCCATGTCGTC), and R9+1 (39191-39175: TGGTCAGAGGATTCGCC).

### Assay for replication initiation from induced cryptic λ prophage

The method is described in [18], only herein, chromosomal DNA was digested with *Nde*I, not *BstE*II.

## Supporting Information

Figure S1 **Aligned conserved sequence regions for 23 lambdoid phages.** Sequence regions were searched using a 33 nt region of sequence similarity between HK620 and λ (“sequence 5” in [79]). The bases in red show greater than 90% sequence homology. The sequence of OOP spans positions -90 (terminator end) through -10 (5′end). The termination sequence for lambda gene *cII*, extending from the left, is at position -33. Position 1 is set as the ATG start for lambda gene *O,* for P22 *orf*48 homologue as *hkaW*, EC_CP1693_21), or a HK097 gp53 homologue *orf*54 (see [Supplementary-material pone.0036498.s002]Fig. S2) [80]. An annotated version of this data was provided in the review [51]. The sequences were obtained and aligned using EBI's implementation of the ClustalW alignment algorithm (http://www.ebi.ac.uk/clustalw/) in full alignment mode as well as a hierarchical clustering method implemented in the Multalin program on the IRNA servers (http://prodes.toulouse.inra.fr/multalin/multalin.html) using a DNA identity matrix and various penalties imposed on gap opening, none on extension. Sequences were obtained from the NCBI nucleotide database. Accession numbers and references are as follows. GI:215104; lambda; *E. coli* [81]. GI:14988; 434; *E. coli* [77]. GI:4539472; 21; *E. coli* [82]. GI:19911589; stx2I; *E. coli* O157:H7 Okayama O-27 [83]. GI:4585377; 933W; *E. coli* O157:H7, strain EDL933 [84]. GI:49523585; phi-4795; *E. coli* strain 4795/95 serotype O84:H4, unpublished. GI:7239813; H-19B; *E. coli* [85]. GI:9634119; HK022; *E. coli* [86]. GI:32128180; Stx2II; *E. coli* O157:H7 Morioka V526 [87]. GI:32128012; Stx1; *E. coli* O157:H7 Morioka V526 [87]. GI:5881592; VT2-Sa; *E. coli* O157:H7 [88]. GI:6901584; HK097; *E. coli* [86]. GI:23343450; Nil2; *E. coli* O157:H7 strain Nil653, unpublished. P22-pbi; *S. enterica serovar typhimurium* [46]. GI:8439576; P22; *S. enterica serovar typhimurium* [89]. GI:1143407; ES18; *S. typhimurium* [90]. GI:13517559; HK620; *E. coli* H strain 2158 [79]. GI:51773702; CP-1639; *E. coli* 1639/77 [91]. GI:24250761; ST64T; *S. enterica serovar typhimurium* [92]. GI:33334157; Sf6; *Shigella flexneri* [93]. GI:14800; Фphi-80; *E. coli* [94]. GI:46357884; ST104T; *S. typhimurium* DT104; phage 434 (GI:14988); phage 21 (GI:4539472); and phage P22 (AF527608.1; GI:21914413; AF217253.1), [95]. Sequence date from this laboratory are shown for: lambda  =  λ*cI*857 (DQ372056), λ*imm*434*cI* (DQ372053.1), λ*imm*21*cI* (DQ372054.1 being revised), and P22-Lambda hybrid  =  λ*cI*857(18,12)P22, representing λhy106 from Dr. S. Hilliker, (DQ372055.1, ); and are expanded and compared to sequences for 434, 21, and P22 in [Supplementary-material pone.0036498.s005]Fig. S5.
(TIF)

Figure S2 **Comparative analysis of lambdoid phage maps.** The regions *cII*-like, *oop*, *orf*, *O-*like and *P*-like are with reference to lambda gene map, e.g., gene *cI* of P22 is equivalent to *cII* of lambda. The numbers in boxes indicate RNA length in nucleotides (nt) for *oop* RNA, or amino acids per proteins cII, Orf, O or P, without specifying the level of gene homology. Color coding relates the similarity of protein length to lambda (pink), P22 (yellow) or Phi 80, with other colors grouping variations based on gene/protein length. Locus identity was obtained using the conserved 33 bp high homology region sequence ([Supplementary-material pone.0036498.s001]Fig. S1) ACTGGATCaATCcACAGGAGTaATTATGaCAAA from the promoter and 5′ end of oop RNA and BLASTed using an expectation value of 1000 and parameters to remove gapping penalty, each containing the conserved sequence with minimum 90% homology: lambda (J02459), 434 (V00635), 21 (AJ237660), Stx2 (AP004402), 933W (AF125520), phi 4795 (AJ556162), H-19B (AF034975), HK022 (NC_002166), Stx2 II (AP005154), Stx 1 (AP005153), VT2-Sa (AP000363), HK097 (AF069529), Nil2 (AJ413274), P22 (AF217253), ES18 (X87420), HK620 (AF335538), CP-1639 (AJ304858), ST64T (AY052766), Sf6 (AF547987), Phi-80 (X13065), and ST104T (AB102868). Examples of the open reading frame left of the O-like protein sequence are orf48 in HK022 [80], and gene p43 in HK97, representing 162 nt (NC_002167). This figure was redrawn with modification from [51].
(TIF)

Figure S3 **Influence of spacing between** ***oop*** **and** ***ori*** **λ on repλ-inhibition.** Influence of spacing between *oop* and *ori*λ on *rep*λ inhibition. A. Plasmid p50 substitutes *E. coli* DNA from the specialized transducing phage λspi156 for the “*ice*” sequence of λ (Table 2) and was made by cloning the 684 bp *Eco*RV-*EcoRI* fragment from λspi156Δ*nin5* [96] into the equivalent sites in pBR322 [69]. B. The stable predicted secondary structures of OOP RNA were obtained using the IDI SciTools OligoAnalyzer 3.0 website. C. EOP of repλ and repP22 phages on host cells with modified Δ*ice oop* ^+^ *ori*λ^+^ plasmids. The averaged data is shown. (Near identical results were seen for each of the plasmids transformed into *E. coli* strain W3350, where standard errors were negligible for the *rep*λ phage, and ranged between <0.1 to 0.28 for the *rep*P22 phage on the different transformed cells.) D. Plasmid modifications to p50: λ DNA fragments in which the DNA interval between *oop* and *ori*λ was varied by deletion or insertion (Table 2).
(TIF)

Figure S4 **Plating-sensitivity to cells exhibiting inhibition phenotype** (**IP**) **and relative plaque size on cell lawns.** A. Variation in susceptibility of *rep*λ phages to the IP. A 0.3 ml aliquot of fresh overnight stationary phase 594[p27R] cells (grown in TB+50 ug/ml Amp) were mixed with 0.1 ml of test phage and 3.0 ml of molten top agar and poured onto a TB plate. Plates were incubated overnight at 30°C and resulting pfu were counted. EOP was calculated as the titer on strain 594[p27R]/titer on 594. The results represent the average of at least two independent assays. Averaged EOP's and standard errors values were: λWT (wild type), 5.17 ×10^−6^ ±2.57×10^−6^; λ*cI*72, 1.73×10^−6^±6.99×10^−7^; λ*imm*434*cI*, 0.01±0.04; λ*imm*21, 0.70±0.06; λ*vir*, 0.41±0.06; λ*cI*90 c17, 1.0×10^−7^±1.0×10^−8^; λ*oR* mutants (*λ*se100a, λse101B, λ109b) 1.15×10^−6^±2.21×10^−7^. Notes: 1) The downstream promoter in λ*cI*90c17 was apparently not strong enough to suppress IP. 2) The plasmids employed in earlier studies [8], [10], [12] inhibited λvir, but each included *cI* repressor gene. We show (Table 4) that λvir was inhibited for plating at 30° in cells with multiple copies of the *O*/*ori*λ plasmid version with *cI* from *imm*λ; whereas, [Supplementary-material pone.0036498.s004]Fig S4A shows λvir is only partially inhibited by cells with *oop* ^+^ *ori*λ^+^ plasmids without *cI*, thus, CI availability to bind *oR* can increase *rep*λ phage sensitivity to IP. B. Portion of λ map showing region of DNA substitution for the *imm*21 and *imm*434 hybrid phages and the portion of λDNA present in plasmids transformed into strain 594. C. Strain 594 was grown overnight to stationary phase in TB [18]; alternatively, 594 transformed with one of the plasmids, shown in part B, was grown overnight in TB+Amp (50 ug/ml). The culture cells (0.25 ml) were mixed with 0.1 ml of phage lysate dilution plus 3 ml TB top agar [18], poured on TB agar plates, and incubated overnight at 30°C. Phage plaque sizes were determined using a tissue culture (inverted) microscope at 4× magnification with an eyepiece grid. Each grid interval was 0.045 mm at 4× magnification. Plaque diameters were measured as grid units, i.e., grids/plaque. Approximately 30 plaques were measured per assay phage on each of the host strains and the average plaque diameter and SE were determined. All assays for a given phage were performed in parallel on each of the host strains using same preparation of agar plates.
(TIF)

Figure S5 **Sequence determination for distal** ***cII-oop*** **to** ***O*** **interval for λ-hybrid** ***imm*** **434,** ***imm*** **21, and** ***rep*** **P22 phages employed.** Hybrid phage sequences compared to λ. The highlighted/underlined bases differ from λ sequence; all data were from this laboratory except sequences for phages 434 and 21; sequence differences rightward from base 38698 are continued in [Supplementary-material pone.0036498.s001]Fig. S1). Phage λ*imm*21, which retains the *rep*λ sequence, had a silent TGC to TGT codon change (not shown in [Supplementary-material pone.0036498.s005]Fig.'s S5 or [Supplementary-material pone.0036498.s001]S1) at 39,033 (one base left of the ITN1 sequence in *O*). Lambda  =  λ*cI*857 (DQ372056) is as in [2]; λ*imm*434*cI* (DQ372053.1); λ*imm*21*cI* (DQ372054.1, being revised); and P22-Lambda hybrid  =  λ*cI*857(18,12)P22, representing λhy106 from Dr. S. Hilliker (DQ372055.1). The comparative partial sequences for non-hybrid phages 434, 21 and P22 were: phage 434 (GI:14988); phage 21 (GI:4539472), and phage P22 (AF527608.1; GI:21914413; AF217253.1).
(TIF)

Figure S6 **PCR assay for plasmid recombination into λ Sip phage within region of λ homology.** PCR Amplification of λ*cI*857 and SIP Phage Isolates 1–4, from Gene *cI* Through Gene *P.* Lanes: 1 & 11, DNA mass ladders from Invitrogen, 2–3, λ*cI*857, 4–5, λ*cI*857 Sip1, 6, λ*cI*857 Sip2, 7–8, λ*cI*857 Sip3, 9–10, λ*cI*857 Sip4. The phages were amplified with primers LMH29 and RPG6 (Methods and Materials). Each PCR was done in duplicate. λ*cI*857 produced the expected 1721 bp fragment. The SIP isolates yielded a 1721 bp fragment, indicating that the p27R plasmid was not integrated into the SIP phage genomes between genes *cI* and *P*.
(TIF)

Figure S7 **Sequenced Sip and Se mutations falling within orf-preX.** A. Organization for transcription of gene *cI* from *pM* and *pE*. Transcription from *pE* is 30–100X the level of transcription from *pM*, [11], [28], [39], [40] and includes an open reading frame preX [14] of 81 codons. Three powerful translational frameshift sites exist within the *cI-rexA-rexB* operon [14], [43] that could influence gene expression from the *pE* promoter, two arise within the N-terminal end of *cI* and one within *rexA* ^1^. B. DNA sequence showing potential translation of preX and its overlap with genes / proteins CI and CRO. This figure shows an alternative interpretation for the position of some Sip mutations shown in Fig. 5, which also map within orf-preX. The previously described Se-mutations confer a *cI* ^-^ phenotype [13]. The mutations se100a and 101b arise in *oR2* and *oR1* between the -35 regions for promoters *pM* and *pR*, and se109b is representative of four other spontaneous se mutations, arising within *oR1* and just left of the -10 region of *pR*. An alternative interpretation is that se100a, se101b and 109b, respectively, confer G56V, T54K and T46N changes in the putative 81 codon preX orf.
(TIF)

Table S1 **EOP of λcI857cro27 on host strains.**
(DOCX)

Table S2 **Relative OOP RNA transcription after prophage induction.**
(DOCX)

Supplemental Methods S1 IP influence on phage plating. Sip phage characterization. Test for plasmid integration. Do λSip phages encode Amp^R^ marker? Plaque PCR of Sip phages. References.
(DOC)
