# Supplementary figures and images for: A CI-Independent Form of Replicative Inhibition: Turn Off of Early Replication of Bacteriophage Lambda
Source: PLoS One. 2012 May 10;7(5):e36498. doi: 10.1371/journal.pone.0036498 (PMC3349717; doi:10.1371/journal.pone.0036498)

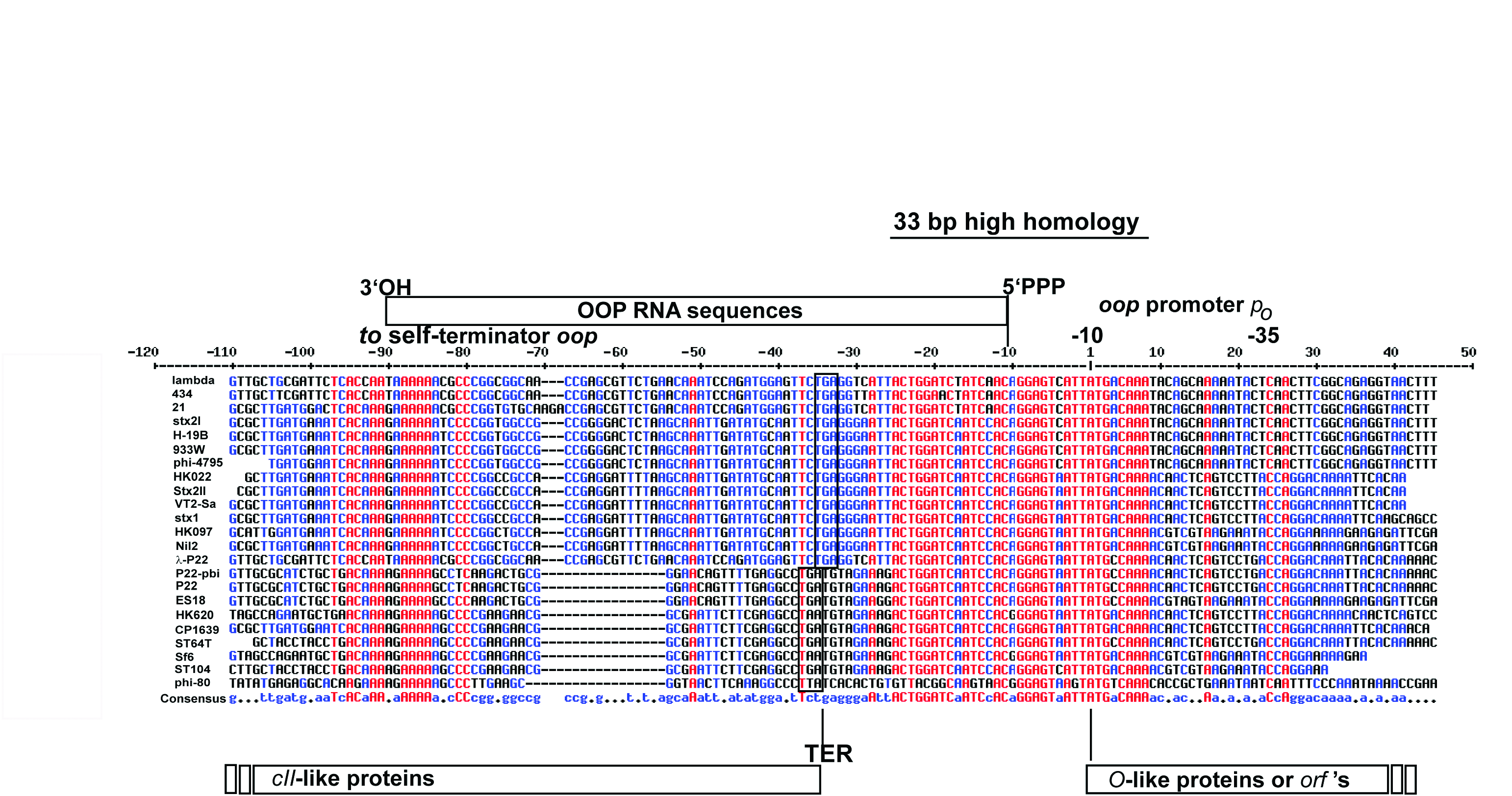

Supplement: Figure S1 — Aligned conserved sequence regions for 23 lambdoid phages. Sequence regions were searched using a 33 nt region of sequence similarity between HK620 and λ (“sequence 5” in [79]). The bases in red show greater than 90% sequence homology. The sequence of OOP spans positions -90 (terminator end) through -10 (5′end). The termination sequence for lambda gene cII, extending from the left, is at position -33. Position 1 is set as the ATG start for lambda gene O, for P22 orf48 homologue as hkaW, EC_CP1693_21), or a HK097 gp53 homologue orf54 (see Fig. S2) [80]. An annotated version of this data was provided in the review [51]. The sequences were obtained and aligned using EBI's implementation of the ClustalW alignment algorithm (http://www.ebi.ac.uk/clustalw/) in full alignment mode as well as a hierarchical clustering method implemented in the Multalin program on the IRNA servers (http://prodes.toulouse.inra.fr/multalin/multalin.html) using a DNA identity matrix and various penalties imposed on gap opening, none on extension. Sequences were obtained from the NCBI nucleotide database. Accession numbers and references are as follows. GI:215104; lambda; E. coli [81]. GI:14988; 434; E. coli [77]. GI:4539472; 21; E. coli [82]. GI:19911589; stx2I; E. coli O157:H7 Okayama O-27 [83]. GI:4585377; 933W; E. coli O157:H7, strain EDL933 [84]. GI:49523585; phi-4795; E. coli strain 4795/95 serotype O84:H4, unpublished. GI:7239813; H-19B; E. coli [85]. GI:9634119; HK022; E. coli [86]. GI:32128180; Stx2II; E. coli O157:H7 Morioka V526 [87]. GI:32128012; Stx1; E. coli O157:H7 Morioka V526 [87]. GI:5881592; VT2-Sa; E. coli O157:H7 [88]. GI:6901584; HK097; E. coli [86]. GI:23343450; Nil2; E. coli O157:H7 strain Nil653, unpublished. P22-pbi; S. enterica serovar typhimurium [46]. GI:8439576; P22; S. enterica serovar typhimurium [89]. GI:1143407; ES18; S. typhimurium [90]. GI:13517559; HK620; E. coli H strain 2158 [79]. GI:51773702; CP-1639; E. coli 1639/77 [91]. GI:24250761; ST64T; S. enterica s [file pone.0036498.s001.tif]

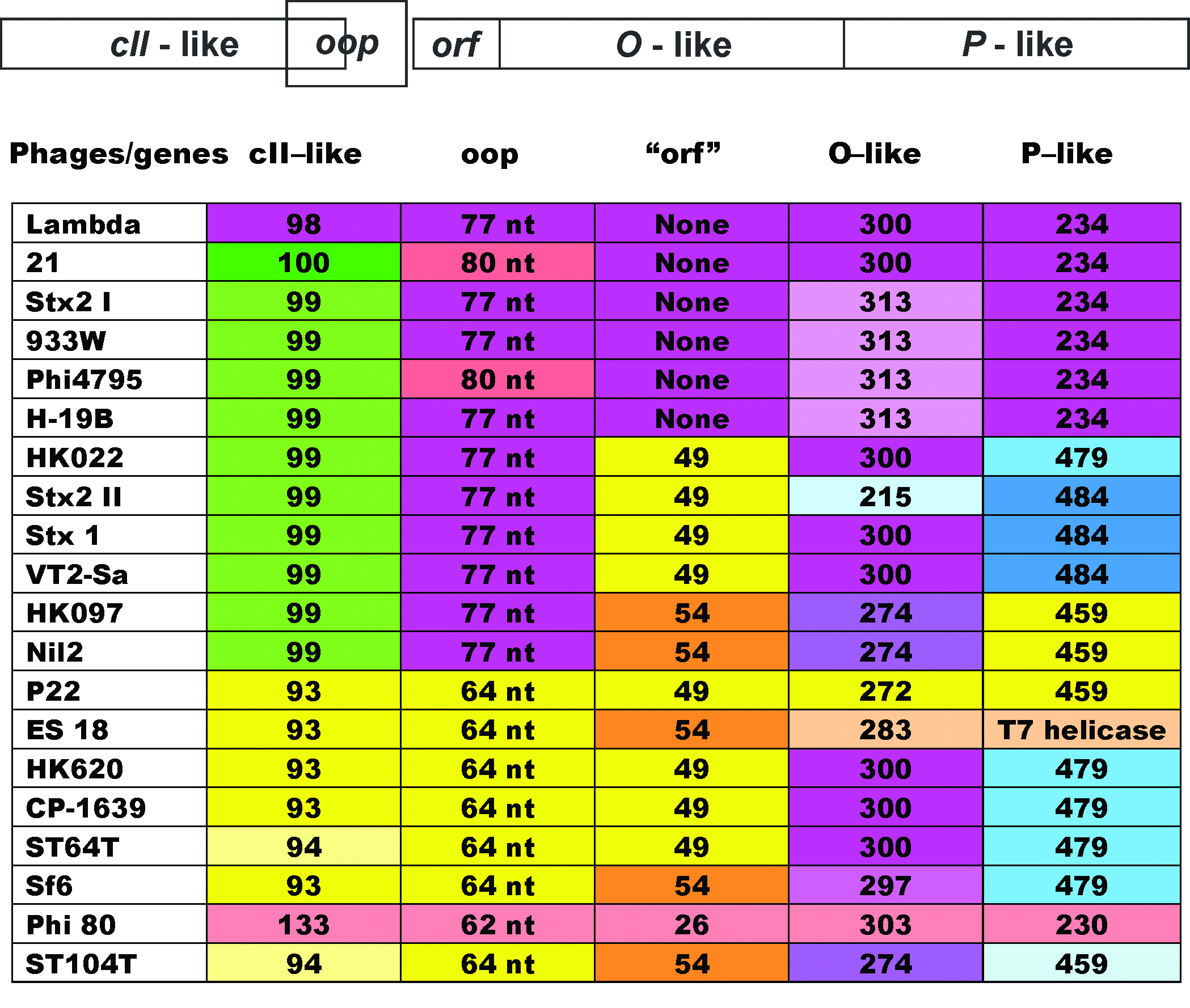

Supplement: Figure S2 — Comparative analysis of lambdoid phage maps. The regions cII-like, oop, orf, O-like and P-like are with reference to lambda gene map, e.g., gene cI of P22 is equivalent to cII of lambda. The numbers in boxes indicate RNA length in nucleotides (nt) for oop RNA, or amino acids per proteins cII, Orf, O or P, without specifying the level of gene homology. Color coding relates the similarity of protein length to lambda (pink), P22 (yellow) or Phi 80, with other colors grouping variations based on gene/protein length. Locus identity was obtained using the conserved 33 bp high homology region sequence (Fig. S1) ACTGGATCaATCcACAGGAGTaATTATGaCAAA from the promoter and 5′ end of oop RNA and BLASTed using an expectation value of 1000 and parameters to remove gapping penalty, each containing the conserved sequence with minimum 90% homology: lambda (J02459), 434 (V00635), 21 (AJ237660), Stx2 (AP004402), 933W (AF125520), phi 4795 (AJ556162), H-19B (AF034975), HK022 (NC_002166), Stx2 II (AP005154), Stx 1 (AP005153), VT2-Sa (AP000363), HK097 (AF069529), Nil2 (AJ413274), P22 (AF217253), ES18 (X87420), HK620 (AF335538), CP-1639 (AJ304858), ST64T (AY052766), Sf6 (AF547987), Phi-80 (X13065), and ST104T (AB102868). Examples of the open reading frame left of the O-like protein sequence are orf48 in HK022 [80], and gene p43 in HK97, representing 162 nt (NC_002167). This figure was redrawn with modification from [51]. (TIF) [file pone.0036498.s002.tif]

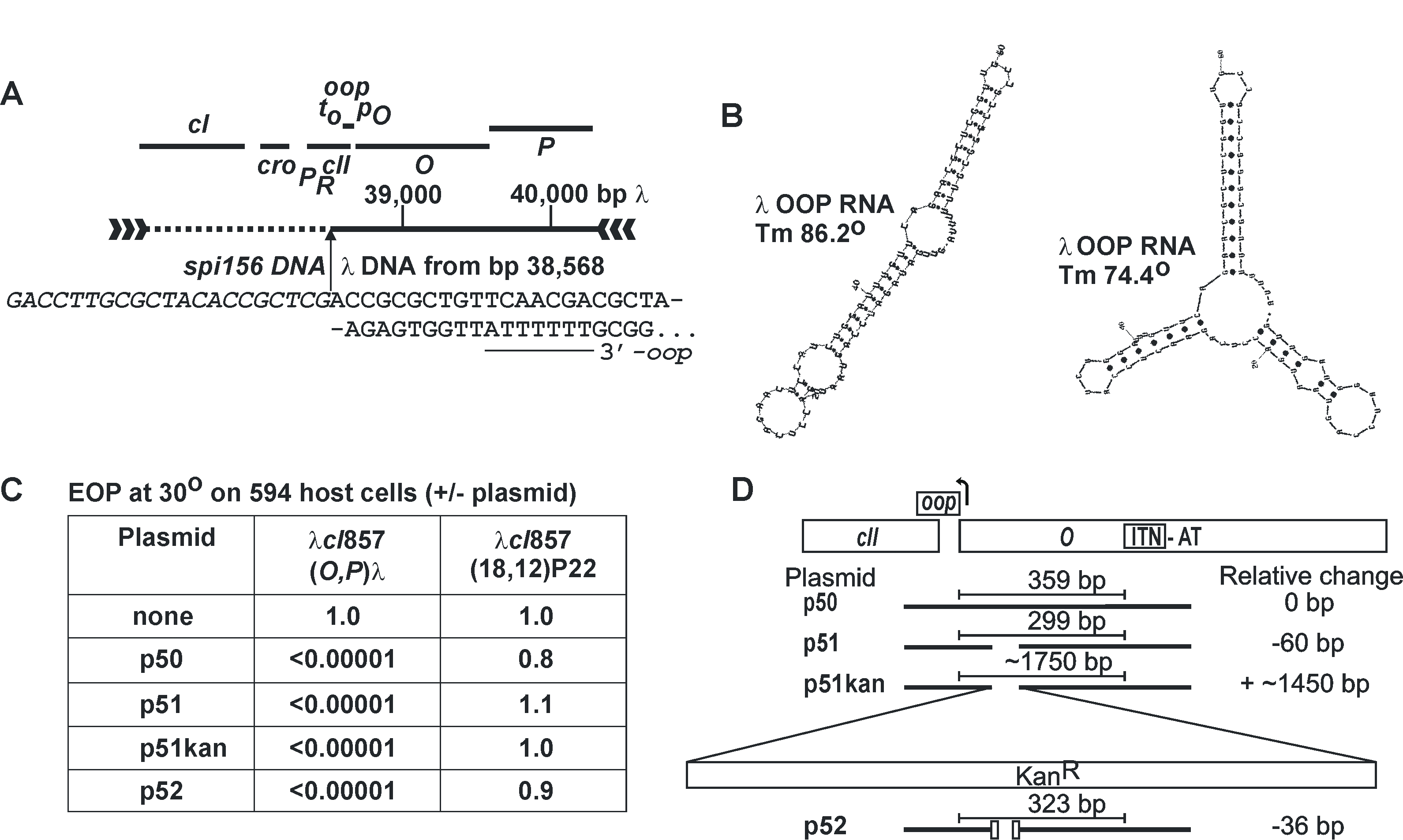

Supplement: Figure S3 — Influence of spacing between oop and ori λ on repλ-inhibition. Influence of spacing between oop and oriλ on repλ inhibition. A. Plasmid p50 substitutes E. coli DNA from the specialized transducing phage λspi156 for the “ice” sequence of λ (Table 2) and was made by cloning the 684 bp EcoRV-EcoRI fragment from λspi156Δnin5 [96] into the equivalent sites in pBR322 [69]. B. The stable predicted secondary structures of OOP RNA were obtained using the IDI SciTools OligoAnalyzer 3.0 website. C. EOP of repλ and repP22 phages on host cells with modified Δice oop + oriλ+ plasmids. The averaged data is shown. (Near identical results were seen for each of the plasmids transformed into E. coli strain W3350, where standard errors were negligible for the repλ phage, and ranged between <0.1 to 0.28 for the repP22 phage on the different transformed cells.) D. Plasmid modifications to p50: λ DNA fragments in which the DNA interval between oop and oriλ was varied by deletion or insertion (Table 2). (TIF) [file pone.0036498.s003.tif]

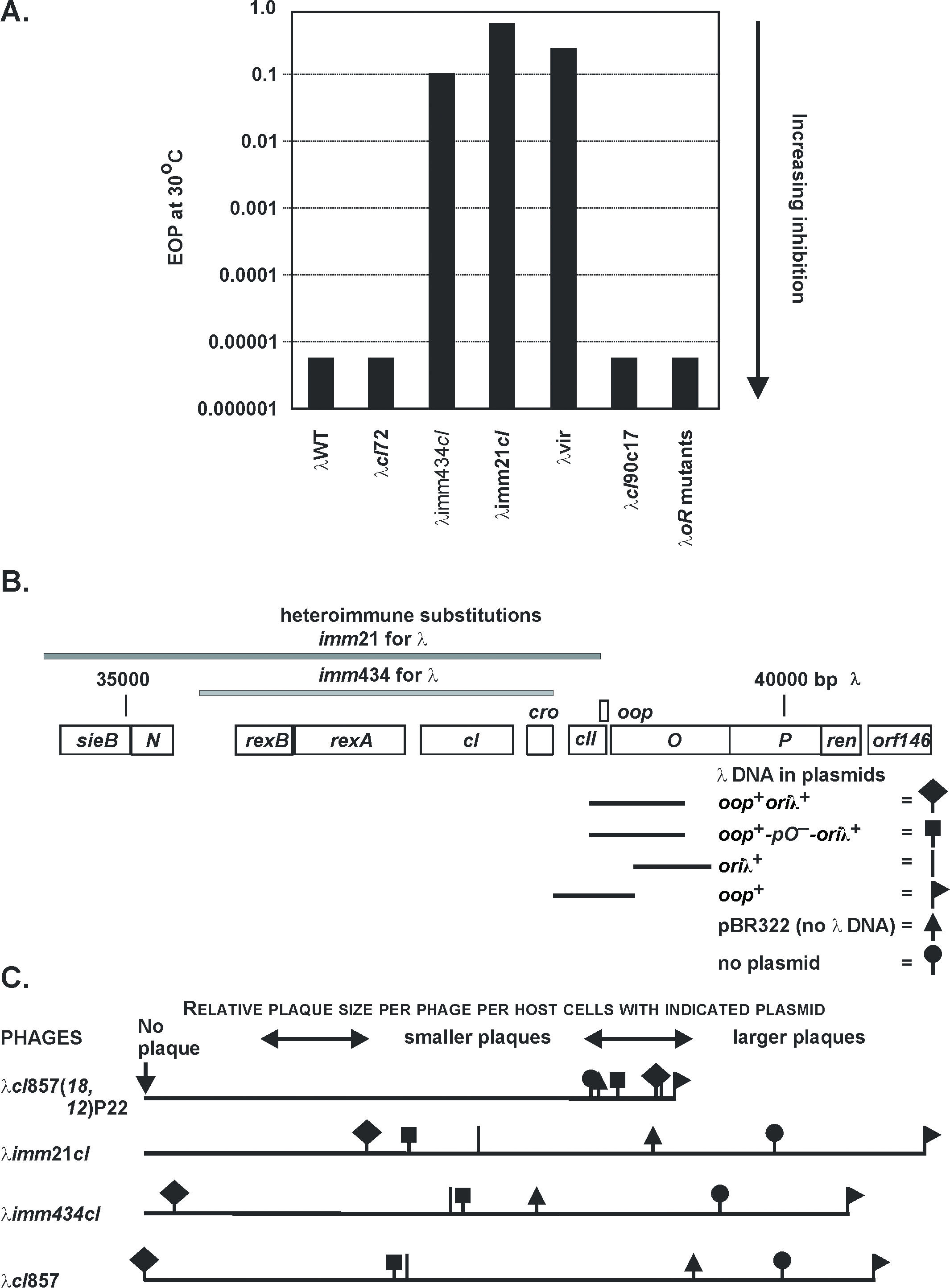

Supplement: Figure S4 — Plating-sensitivity to cells exhibiting inhibition phenotype (IP) and relative plaque size on cell lawns. A. Variation in susceptibility of repλ phages to the IP. A 0.3 ml aliquot of fresh overnight stationary phase 594[p27R] cells (grown in TB+50 ug/ml Amp) were mixed with 0.1 ml of test phage and 3.0 ml of molten top agar and poured onto a TB plate. Plates were incubated overnight at 30°C and resulting pfu were counted. EOP was calculated as the titer on strain 594[p27R]/titer on 594. The results represent the average of at least two independent assays. Averaged EOP's and standard errors values were: λWT (wild type), 5.17 ×10^−6^ ±2.57×10^−6^; λcI72, 1.73×10^−6^±6.99×10^−7^; λimm434cI, 0.01±0.04; λimm21, 0.70±0.06; λvir, 0.41±0.06; λcI90 c17, 1.0×10^−7^±1.0×10^−8^; λoR mutants (λse100a, λse101B, λ109b) 1.15×10^−6^±2.21×10^−7^. Notes: 1) The downstream promoter in λcI90c17 was apparently not strong enough to suppress IP. 2) The plasmids employed in earlier studies [8], [10], [12] inhibited λvir, but each included cI repressor gene. We show (Table 4) that λvir was inhibited for plating at 30° in cells with multiple copies of the O/oriλ plasmid version with cI from immλ; whereas, Fig S4A shows λvir is only partially inhibited by cells with oop + oriλ+ plasmids without cI, thus, CI availability to bind oR can increase repλ phage sensitivity to IP. B. Portion of λ map showing region of DNA substitution for the imm21 and imm434 hybrid phages and the portion of λDNA present in plasmids transformed into strain 594. C. Strain 594 was grown overnight to stationary phase in TB [18]; alternatively, 594 transformed with one of the plasmids, shown in part B, was grown overnight in TB+Amp (50 ug/ml). The culture cells (0.25 ml) were mixed with 0.1 ml of phage lysate dilution plus 3 ml TB top agar [18], poured on TB agar plates, and incubated overnight at 30°C. Phage plaque sizes were determined using a tissue culture (inverted) microscope at 4× magnification with an eyepiece grid. Each grid int [file pone.0036498.s004.tif]

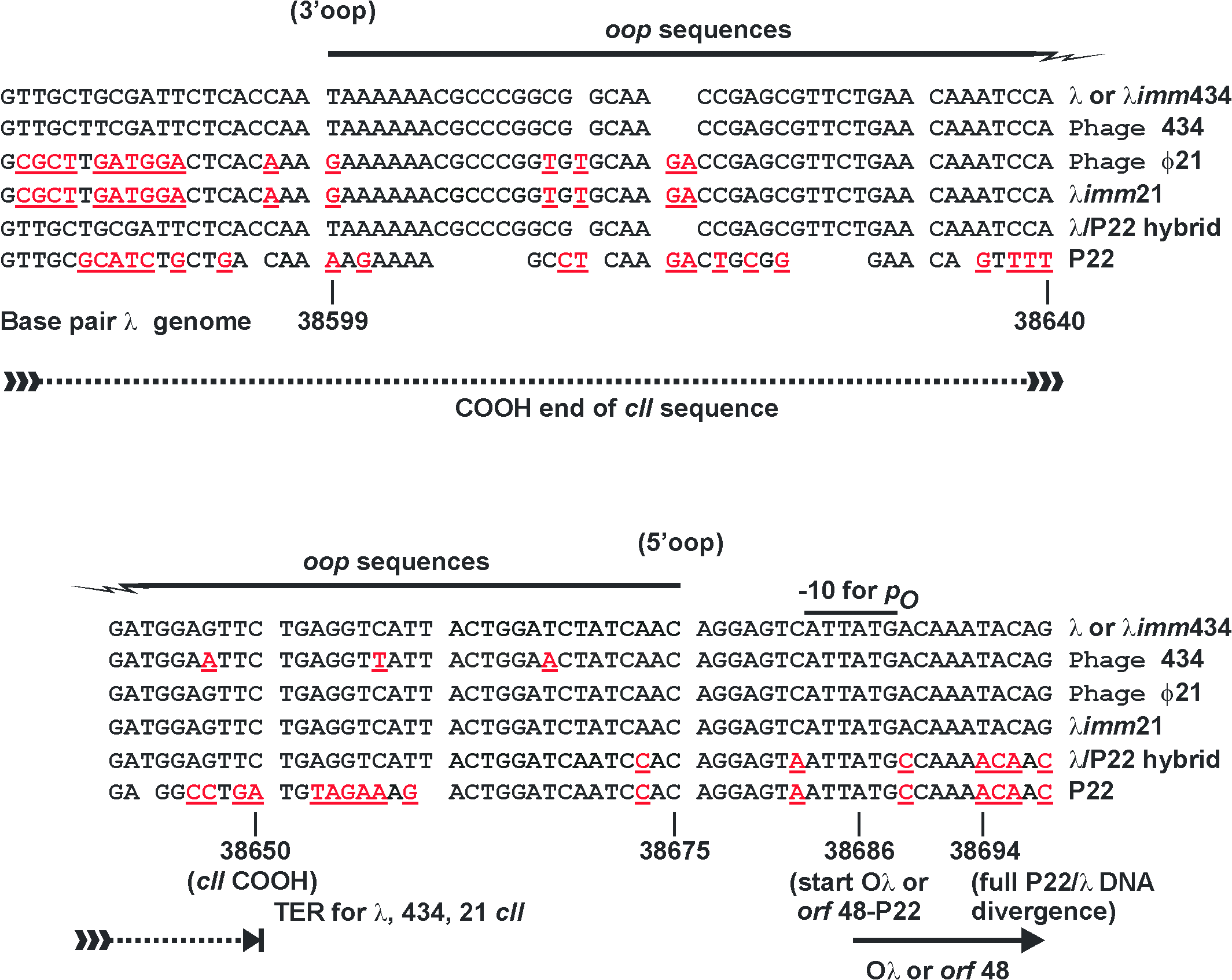

Supplement: Figure S5 — Sequence determination for distal cII-oop to O interval for λ-hybrid imm 434, imm 21, and rep P22 phages employed. Hybrid phage sequences compared to λ. The highlighted/underlined bases differ from λ sequence; all data were from this laboratory except sequences for phages 434 and 21; sequence differences rightward from base 38698 are continued in Fig. S1). Phage λimm21, which retains the repλ sequence, had a silent TGC to TGT codon change (not shown in Fig.'s S5 or S1) at 39,033 (one base left of the ITN1 sequence in O). Lambda = λcI857 (DQ372056) is as in [2]; λimm434cI (DQ372053.1); λimm21cI (DQ372054.1, being revised); and P22-Lambda hybrid = λcI857(18,12)P22, representing λhy106 from Dr. S. Hilliker (DQ372055.1). The comparative partial sequences for non-hybrid phages 434, 21 and P22 were: phage 434 (GI:14988); phage 21 (GI:4539472), and phage P22 (AF527608.1; GI:21914413; AF217253.1). (TIF) [file pone.0036498.s005.tif]

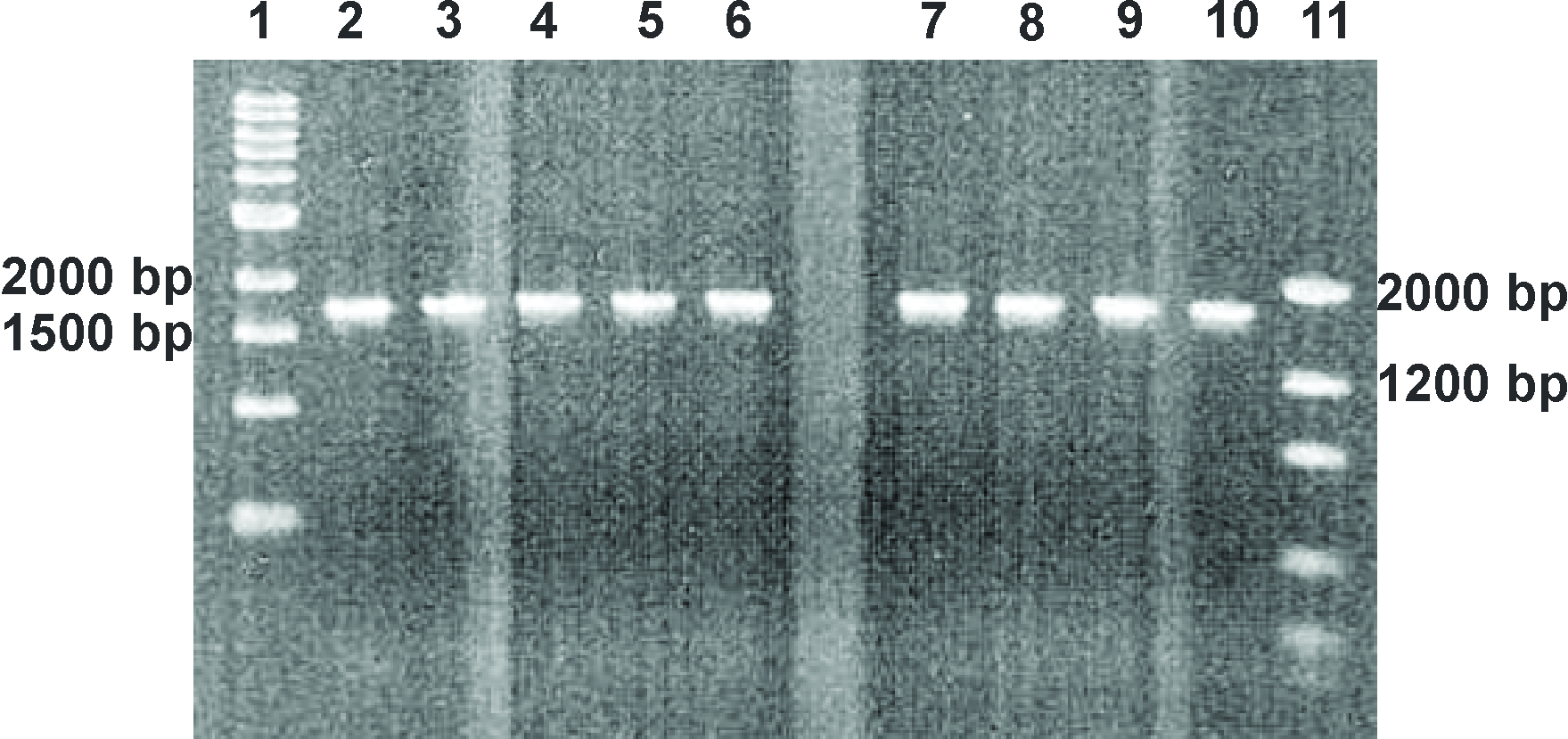

Supplement: Figure S6 — PCR assay for plasmid recombination into λ Sip phage within region of λ homology. PCR Amplification of λcI857 and SIP Phage Isolates 1–4, from Gene cI Through Gene P. Lanes: 1 & 11, DNA mass ladders from Invitrogen, 2–3, λcI857, 4–5, λcI857 Sip1, 6, λcI857 Sip2, 7–8, λcI857 Sip3, 9–10, λcI857 Sip4. The phages were amplified with primers LMH29 and RPG6 (Methods and Materials). Each PCR was done in duplicate. λcI857 produced the expected 1721 bp fragment. The SIP isolates yielded a 1721 bp fragment, indicating that the p27R plasmid was not integrated into the SIP phage genomes between genes cI and P. (TIF) [file pone.0036498.s006.tif]

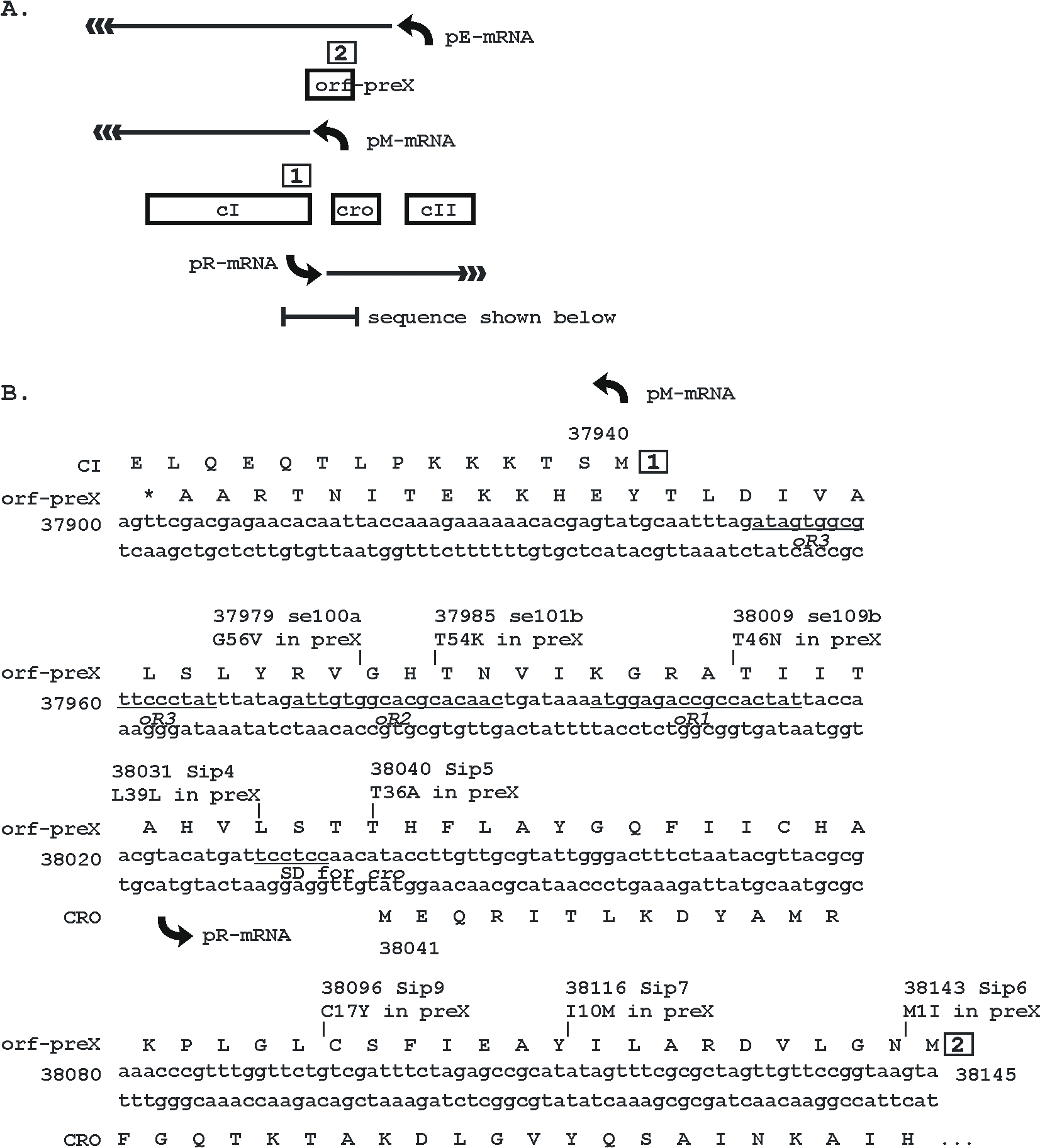

Supplement: Figure S7 — Sequenced Sip and Se mutations falling within orf-preX. A. Organization for transcription of gene cI from pM and pE. Transcription from pE is 30–100X the level of transcription from pM, [11], [28], [39], [40] and includes an open reading frame preX [14] of 81 codons. Three powerful translational frameshift sites exist within the cI-rexA-rexB operon [14], [43] that could influence gene expression from the pE promoter, two arise within the N-terminal end of cI and one within rexA 1. B. DNA sequence showing potential translation of preX and its overlap with genes / proteins CI and CRO. This figure shows an alternative interpretation for the position of some Sip mutations shown in Fig. 5, which also map within orf-preX. The previously described Se-mutations confer a cI - phenotype [13]. The mutations se100a and 101b arise in oR2 and oR1 between the -35 regions for promoters pM and pR, and se109b is representative of four other spontaneous se mutations, arising within oR1 and just left of the -10 region of pR. An alternative interpretation is that se100a, se101b and 109b, respectively, confer G56V, T54K and T46N changes in the putative 81 codon preX orf. (TIF) [file pone.0036498.s007.tif]
