# Supplementary material for: A CI-Independent Form of Replicative Inhibition: Turn Off of Early Replication of Bacteriophage Lambda
Source: PLoS One. 2012 May 10;7(5):e36498. doi: 10.1371/journal.pone.0036498 (PMC3349717; doi:10.1371/journal.pone.0036498)
Supplement: Table S1 — EOP of λcI857cro27 on host strains. (DOCX) [file pone.0036498.s008.docx]

**Table S1. EOP of λ*cI*857*cro*27 on host strains ^a^**

| **Host Cells** | **Plating** | **Phage Plaques** | | | | **Average EOP** |
| --- | --- | --- | --- | --- | --- | --- |
|  | **Temp.** | **10^-3^** | **10^-7^** | **10^-8^** | **Titer** |  |
| **TC600** | **39^o^C** | **^--^** | **170** | **19** | **1.8 x 10^9^** | **1.0** |
| **TC600** | **30^o^** | **0 ^b^** | **0** |  | **<10^3^** | **<5.6 x 10^-7^** |
| **594[p27]** | **30^o^** | **0** | **0** |  | **<10^3^** | **<5.6 x 10^-7^** |
| **594[p27R]** | **30^o^** | **0** | **0** |  | **<10^3^** | **<5.6 x 10^-7^** |
| **594[*p27RpO*^-^]** | **30^o^** | **0 ^b^** | **0** |  | **<10^3^** | **<5.6 x 10^-7^** |
| **594[p27RΔAT]** | **30^o^** | **0** | **0** |  | **<10^3^** | **<5.6 x 10^-7^** |
| **594[p27R-R45OOP]** | **30^o^** | **0 ^b^** | **0** |  | **<10^3^** | **<5.6 x 10^-7^** |
| **594[p27RΔINT 1-4]** | **30^o^** | **0** | **0** |  | **<10^3^** | **<5.6 x 10^-7^** |
| **594[p28]** | **30^o^** | **0** | **0** |  | **<10^3^** | **<5.6 x 10^-7^** |
| **594[p29]** | **30^o^** | **0 ^b^** | **0** |  | **<10^3^** | **<5.6 x 10^-7^** |

**^a^** Strains in lines 3,4,6 express inhibition phenotype. The experiment was to determine if moving the *cro*27 mutation into λ*cI*857 confers a Sip plating phenotype, capable of escaping IP. Plasmid strains were first streaked onto LBamp_50_ agar plates (as TB agar plates, but with 5g/l Bacto yeast extract) and grown at 30^o^C overnight. Individual colonies were picked and streaked again on a 30^o^C LBamp_50_ master plate. Cells of each strain were then inoculated from the 30^o^C master plates into 20ml of Luria Broth (as TB, but with 5g/l Bacto yeast extract) plus 50mg/ml ampicillin. The TC600 strain, which carries SupE suppressor, was grown on LB without ampicillin, and supports λ plating as efficiently as does strain 549 at all temperatures. The cultures were grown at 30^o^C to saturation. The λ*cI*857*cro*27 phage was diluted in Φ80 buffer, pH7.6, and 0.1ml aliquots combined with 0.25ml of the overnight culture of each strain plus 2.5ml of top agar and poured onto LB agar plates. The phage was plated at 10^-3^ and 10^-7^ plating dilutions on each of host strains and on TC600 at 30^o^C. The phage was plated at 10^-7,-8^ on TC600 @ 39^o^C as a control for actual phage titer. Plates were incubated for 24 hours and plaques numbers determined.

**^b^** Some cell lawn killing, but no visible plaques.
