# Supplementary material for: A CI-Independent Form of Replicative Inhibition: Turn Off of Early Replication of Bacteriophage Lambda
Source: PLoS One. 2012 May 10;7(5):e36498. doi: 10.1371/journal.pone.0036498 (PMC3349717; doi:10.1371/journal.pone.0036498)
Supplement: Table S2 — Relative OOP RNA transcription after prophage induction. (DOCX) [file pone.0036498.s009.docx]

**Table S2. Relative OOP RNA transcription after prophage induction^a^**

| **Minutes after de-repression^b^** | **Defective *cI*857 prophage^c^** | **λ*cI*857 *S*am7^d^** |
| --- | --- | --- |
| **0** | **2** | **nd^e^** |
| **3** | **~0** | **~0** |
| **5** | **~0** | **~0** |
| **15** | **33** | **40** |
| **21** | **51** | **64** |
| **25** | **52** | **nd** |
| **31** | **nd** | **54** |
| **41** | **48** | **33** |
| **56** | **40** | **28** |
| **71** | **42** | **6** |
| **86** | **41** | **3** |

^a^ Results from data in Fig. 3 in [reference 2, Supplemental Methods S1] resulting from pulse labeling cultures for one minute with uridine -5-^3^H.

^b^ Culture cells shifted from 30^o^ to 41^o^ where prophage CI[Ts]857 repressor is denatured.

^c^ Strain SA431 with chromosomally integrated, defective nonexcisable prophage with Δ431 removing the late lysis and morphogenesis genes as was described for strain Y836 (Table 1).

^d^ The prophage is integrated within a nonpermissive strain where the *S*am7 mutation is not suppressed, resulting in a buildup of mature phage particles within cells without lysis.

^e^ nd, not assayed.
