# Supplementary material for: A CI-Independent Form of Replicative Inhibition: Turn Off of Early Replication of Bacteriophage Lambda
Source: PLoS One. 2012 May 10;7(5):e36498. doi: 10.1371/journal.pone.0036498 (PMC3349717; doi:10.1371/journal.pone.0036498)
Supplement: Supplemental Methods S1 — IP influence on phage plating. Sip phage characterization. Test for plasmid integration. Do λSip phages encode AmpR marker? Plaque PCR of Sip phages. References. (DOC) [file pone.0036498.s010.doc]

**Supplemental Methods S1**

**IP influence on phage plating.** Plasmids derived from pBR322 that included the DNA segments *oop-ori*λ, *oop*, *ori*λ, or *oop p_O_***^–^***-ori*λ synthetically inactivated for the *p_O_* promoter for *oop* (Fig. 1A) were transformed into the prototrophic *E. coli* strain 594 Sup^o^ F^-^ *lac*3350 *gal*K2 *galT*22. Phage plaque sizes on host cells were measured as described in legend for Fig. S4.

**Sip phage characterization.** We examined if the sip mutants carried the *oop-ori*λ plasmid. The p27R plasmid contains homologous λ DNA from 38517 to 39175 (from *ice* in gene *cII* through *ori*λ in gene *O*). Its incorporation into a lambda genome of 48502 base pairs would be theoretically possible, generating a 51375 base pair λ genome. Lambda is reported as being capable of packaging between 78 and 105% of its genome , *i.e.* up to about 51 kilobases. A phage containing this plasmid recombined within its genome would contain three functional origins for replication initiation, its own *ori*λ, and both *ori*λ and ColE1 origins on the plasmid. The ColE1 origin could suppress phage replication if initiation from *ori*λ was prevented. We examined if sip phages grown on 594 cells could bypass replication initiation from *ori*λ. The sip mutants were unable to plate on *dnaB*grpD55 host cells, which supported pBR322 replication and plasmid maintenance between 25 and 42^o^C, the presence or absence of active *rom* protein in the *oop-ori*λ plasmid made no difference, i.e. λ*cI*857 was severely inhibited (EOP <0.00001) from plating, but all sip mutants plated on 594 cells with these plasmid at high efficiency (EOP 0.25 – 1, large plaques). Phage lysates made from single plaque isolates of the sip phages prepared from twice-stripped single plaque lysates on 594 host cells (without plasmid) retained the sip phenotype, and lysogens made with these lysates did not encode resistance to amplicillin (i.e. did not carry β-lactamase gene from plasmid). In combination, these results support the conclusion that the sip mutations mapping in *orf-*preX, the 18 base leader sequence between the transcriptional start from *p_R_* promoter and start of *cro* gene, in *cro* or within *O* can suppress the inhibition phenotype.

**Test for plasmid integration.** λ*cI*857 and SIP isolates 1-4 were amplified using PCR primers LMH29 (λ37905-37922 and RMH25 (λ39626-39609**)** to amplify the phage genomes from the N-terminal region of *cI* through the N-terminal region of *P*. These two primers bind outside the region of sequence homology between p27R and the λ genome. In the absence of a p27R plasmid insertion, the phage 1721 nt PCR fragment was expected. A SIP phage containing an inserted p27R plasmid would produce a PCR fragment of 4521 nt. The single plaque PCR products were visualized by running on an agarose gel. All amplified phages, λ*cI*857 and SIP isolates 1-4, produced PCR fragments of approximately 1721 bp, with no visible larger fragments, suggesting that the p27R plasmid had not been integrated within the SIP phage genomes (at least not between phage genes *cI* and *P*).

**Do λSip phages encode Amp^R^ marker?** Determining if 10 λ*cI*857Sip mutants had acquired by recombination the p27R[Amp^R^] plasmid during the selection for spontaneous Sip mutants of λc1857 on 594[p27R] host. Sip 1-10 phage lysates that were prepared on 594 host cells were plated at dilutions of 10^-2,-5,-6^ on 594. 0.1ml of phage lysate dilution was combined with 0.25ml overnight culture of 594 cells plus 2.5ml of top agar and poured onto a) TB agar plates and b) duplicates on TBamp_50_ agar plates. The plates were incubated overnight at 30^o^C. The following day phage plaques were counted and incubation was continued for 48 hr at 30^o^C and Amp^R^ cfu, arising within the plaques, or anywhere on the plates, assessed. As a control, 0.1ml of direct phage lysate from 10^-5^ dilution was spread on TB agar plates to determine if any cell contamination was observed. No Amp^R^ cfu were observed after 48 hr incubation at 30^o^C on any of the assay or control plates, indicating that the frequency of phages within the λ*cI*857 Sip lysates that had integrated the Amp^R^ p27R plasmid was <10^-5^.

**Plaque PCR of Sip phages.** Single plaque isolates were stripped on sensitive 594 cells, incubated overnight. About 1mm plaque was picked using sterile pipet tip to 50ul TE* buffer (10mM Tris, 0.1mM Na2EDTA, pH 7.6), vortexed lightly, incubated 1 hr at 37^o^C, heated for 5 min at 96^o^C in thermocycler, centrifuged at 13,000g in Eppendorf Microfuge, and 40 ul of plaque eluate was removed. 10 ul of the eluate was used in a 100ul PCR reaction with 100pmol of each primer and nucleoside triphosphates each at 1.25 mM.
